# The Effect of Relative Humidity in Conductive Atomic Force Microscopy

**DOI:** 10.1002/adma.202405932

**Published:** 2024-09-11

**Authors:** Yue Yuan, Mario Lanza

**Affiliations:** ^1^ Materials Science and Engineering Program Physical Science and Engineering Division King Abdullah University of Science and Technology (KAUST) Thuwal 23955 Saudi Arabia; ^2^ Department of Materials Science and Engineering National University of Singapore Singapore 117575 Singapore

**Keywords:** 2D materials, conductive atomic force microscopy, metrology, nanoelectronics, relative humidity

## Abstract

Conductive atomic force microscopy (CAFM) analyzes electronic phenomena in materials and devices with nanoscale lateral resolution, and it is widely used by companies, research institutions, and universities. Most data published in the field of CAFM is collected in air at a relative humidity (RH) of 30–60%. However, the effect of RH in CAFM remains unclear because previous studies often made contradictory claims, plus the number of samples and locations tested is scarce. Moreover, previous studies on this topic did not apply current limitations, which can degrade the CAFM tips and generate false data. This article systematically analyzes the effect of RH in CAFM by applying ramped voltage stresses at over 17,000 locations on ten different samples (insulating, semiconducting, and conducting) under seven different RH. An ultra‐reliable setup with a 110‐pA current limitation during electrical stresses is employed, and excellent CAFM tip integrity after thousands of tests is demonstrated. It is found that higher RH results in increased currents due to the presence of a conductive water meniscus at the tip/sample junction, which increases the effective area for electron flow. This trend is observed in insulators and ultra‐thin semiconductors; however, in thicker semiconductors the electron mean free path is high enough to mask this effect. Metallic samples show no dependence on RH. This study clarifies the effect of relative humidity in CAFM, enhances understanding of the technique, and teaches researchers how to improve the reliability of their studies in this field.

## Graphical abstract

In this article, the impact of relative humidity (RH) on conductive atomic force microscopy (CAFM) measurements is investigated. For insulating and ultra‐thin (less than 5 nm) semiconducting samples, a RH at 4% results in lower and more inhomogeneous currents compared to RH = 54%. In contrast, for thicker semiconducting (above 5 nm) and metallic samples, RH has no significant effect. These observations are confirmed by over 17,000 tests at different locations of ten samples.

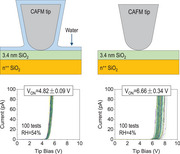

## 1 Introduction

Conductive atomic force microscopy (CAFM) has emerged as a powerful technique that allows measuring the electrical properties of different types of materials at the nanoscale with high lateral resolution, typically down to ≈1 nm^[^ ^1^ ^]^ although recent studies have demonstrated atomic resolution.^[^ ^2^ ^]^ In its origin, CAFM was conceived to analyze the tunneling current across thin dielectric films,^[^ ^3^ ^]^ but with time its use has expanded to many other applications, including piezoelectricity, photovoltaics, crystallography, and lithography, among many others.^[^ ^4^ ^]^ The working principle of CAFM consists of a conductive nanoprobe (also called CAFM tip) located at the end of a bendable cantilever that is brought into contact with the material under test using very precise actuators in the three directions of space (X, Y, Z).^[^ ^1^ ^]^ The CAFM tip has a radius at its apex (*R_tip_*) that typically ranges between 2 and 200 nm, which leads to a very small tip/sample contact area (*A_c_*) of 1–600 nm^2^.^[^ ^5^ ^]^ When the tip/sample system is excited (normally by applying an external voltage), electrical current can flow at the junction, and this can be monitored using a current‐to‐voltage preamplifier, which gives information about the electrical properties of the sample at that specific location.^[^ ^1^ ^]^ Some samples can be excited using other energy sources, such as light or mechanical strain.^[^ ^4^ ^]^

If the junction between the CAFM tip and the sample is completely clean, the current registered by the CAFM tip equals *I* = *A_eff_* × *J*, where *A_eff_* is the effective area across which electrons can flow, and *J* is the average current density across *A_eff_*. When the CAFM tip is placed on the surface of an insulating material with negligible lateral conductivity, *A_eff_* is equal to *A_c_*.^[^ ^1^ ^]^ When the CAFM tip is placed on the surface of a semiconducting material, the lateral conductivity is not zero, and the value of *A_eff_* increases significantly according to the electron mean free path (*l*) in the semiconductor, and it can be calculated as *A_eff_* = π × *l* ^2^.^[^ ^6^ ^]^ When the CAFM tip is placed on the surface of a metallic material (which has a very high lateral conductivity) the value of *A_eff_* equals the whole area covered by the metal, and it can reach values higher than 10,000 µm^2^.^[^ ^7^ ^]^ However, in ultra‐thin conductive materials like graphene the value of *A_eff_* is also linked to the electron mean free path.^[^ ^8^ ^]^

When using CAFM, one can apply ramped voltage stresses (RVS) at a given location and register the current; the resulting current versus voltage (*I*–*V*) plots give important information about the material, such as: i) the onset potential (*V_ON_*), which is defined as the minimum voltage at which current above the noise level (typically ≈1 pA) can be detected; and ii) the slope of the *I*–*V* plot, from which the conduction mechanism can be deduced.^[^ ^9^, ^10^ ^]^ If RVS is applied at multiple locations of the sample, one can analyze how these magnitudes change from one point to another, which gives information about the homogeneity of the sample.^[^ ^11^ ^]^ In that direction, even more powerful is to apply a constant voltage and move the tip laterally to scan a given area of the sample. This allows creating of a 3D current map of the sample, in which each pixel of the image reveals the current driven by the sample at each location.^[^ ^1^ ^]^ For example, a current map formed by 256 pixels per line and 256 lines contains information about 65,536 locations, although only at one specific voltage. Moreover, both RVS and current maps can be repeated multiple times at the same location of the sample to assess how the currents evolve over stress time, which has allowed the detection of multiple phenomena, such as random telegraph noise,^[^ ^12^ ^]^ stress‐induced leakage current,^[^ ^13^ ^]^ dielectric breakdown,^[^ ^14^ ^]^ and resistive switching,^[^ ^15^ ^]^ among many others. An additional advantage of CAFM is that the bending of the cantilever can be measured simultaneously and independently using an optical or piezoresistive sensor,^[^ ^1^ ^]^ giving information about the contact force (*F_c_*) between the tip and the sample (during RVS) and/or about the topography of the sample (during lateral scans).

However, the data registered by the CAFM can be severely affected by the relative humidity (RH) of the environment in an uncertain manner. In particular, as the CAFM tip approaches the sample, the water layers present on the surfaces of both tip and sample form a water meniscus due to the capillary condensation,^[^ ^16^ ^]^ leading to changes in surface charge, electrical field distribution, and adhesion force.^[^ ^17^, ^18^ ^]^ These effects can be especially pronounced when measuring ultra‐thin materials in which surface interactions and number of dangling bonds play a dominant role in determining electrical properties. A few studies^[^ ^18^, ^19^, ^20^, ^21^ ^]^ have tried to elucidate the effect of RH in CAFM experiments, but none of them was systematic: the amount of data presented and the number of samples analyzed was quite limited. Moreover, in many cases the data shown might have been affected by CAFM tip degradation because: i) no current limitation was applied during the electrical measurements, which can easily damage both the tip and the sample;^[^ ^22^, ^23^ ^]^ and ii) the good conductivity of the CAFM tip after the experiments was not demonstrated. In fact, some of these studies presented opposing claims. For example, in refs. [18] the authors claimed that CAFM measurements carried out at higher RH result in higher currents because the water meniscus surrounding the tip/sample junction increases *A_eff_*, while in refs. [19, 20, 21] the authors claimed that the currents are lower at higher RH due to the presence of contaminants and/or molecules of water between the tip and the sample. Overall, the effect of RH in CAFM measurements is a very important topic that is still not understood.

In this article, we use an ultra‐reliable measuring protocol to systematically analyze the effect of RH in CAFM experiments. We measure current‐limited RVS under seven different RH levels (54, 46, 40, 30, 20, 10, and 4%) at thousands of randomly selected locations of ten different samples, including insulating, semiconducting and conducting materials, which are: 1.5, 2.3, 3.4 and 5.6 nm SiO_2_ prepared by thermal oxidation of n^++^Si, 3.32 nm 2D layered hexagonal boron nitride (h‐BN) flakes produced by mechanical exfoliation and transferred on a Ru substrate, 1.37 and 5.2 nm 2D layered h‐BN films grown by chemical vapor deposition (CVD) on Cu, 1.88 and 8.66 nm 2D layered molybdenum disulfide (MoS_2_) flakes produced by mechanical exfoliation and transferred on a Ru substrate, and highly conductive 10 nm Ru films on a Ta/SiO_2_/Si substrate.

## 2 The CAFM Setup

The process followed to fabricate each sample is described in the Methods section. All the samples are characterized in a Bruker Dimension Icon CAFM by applying RVS at different randomly selected locations in a 10 × 10 matrix. The minimum distance between each testing location is kept at 1 µm to avoid point‐to‐point interactions. When measuring the semiconducting and the insulating samples, positive RVS is applied to the CAFM tip while keeping the substrate of the sample grounded. In this way, electrons are always injected from the substrate of the sample and undesired electrochemistry related to the water meniscus (local anodic oxidation) is avoided.^[^ ^24^ ^]^ The voltage is ramped from 0 to 10 V, although the RVS is stopped when the current surpasses 110 pA to prevent CAFM tip degradation.^[^ ^23^ ^]^ When measuring conducting samples, RVS ranging from −1.5 to +1.5 mV are applied to the CAFM tip (sample holder grounded), with a current sensitivity of 20 pA V^−1^ where the current is saturated to ≈200 pA (although in this case the RVS is not stopped). The RH of the chamber can be reduced to 4% by inserting nitrogen gas through an inlet; more details about the setup are given in the Methods section. It should be noted that complete removal of the water meniscus at RH = 4% (without heating the surface of the sample) is improbable;^[^ ^25^ ^]^ hence here we are not claiming that at RH = 4% the water meniscus has been completely removed, we think that its size has been extremely reduced.

For higher reliability, we used two types of CAFM tips. The first one consists of solid Pt CAFM tips (RMN 25PT300B), which feature nominal spring constant (*k*) of 18 N m^−1^ and *R_tip_* = 25 nm, although scanning electron microscopy (SEM) measurements collected in ten new tips reveal that the real value of *R_tip_* ranges from 10.9 and 28.5 nm.^[^ ^22^ ^]^ The second one consists of CONTV‐PT tips, which have a Si body coated with 20 nm Pt and feature *k* = 0.2 N m^−1^ and *R_tip_* = 25 nm, although SEM measurements in five new CONTV‐PT tips reveal real *R_tip_* values ranging between 20.7 and 28.9 nm.^[^ ^22^ ^]^ In general, it is widely accepted that solid Pt tips are more durable;^[^ ^22^ ^]^ however, their shape is more irregular, they are more expensive, and so far fewer authors have used them–because this type of tip is newer. Therefore, in this study some samples were characterized with both types of CAFM tips, and the results were compared.

When the same sample is measured with different CAFM tips, slightly different values of current can be obtained, even when using tips of the same type and box under identical parameters (see Figure S1, Supporting Information). The reason is the inherent variability of *k* and *R_tip_* from one tip to another (tolerances up to ±40% are indicated by the manufacturer).^[^ ^26^ ^]^ One could measure the real value of *k* for different tips and ensure that the experiments are being carried out under identical *F_c_*, but even in such case, the data collected with different tips will slightly differ due to the remaining variability provoked by *R_tip_*, which is an uncontrollable parameter of the setup. One could try to be even more accurate and calculate *A_c_* by measuring *R_tip_* via SEM and using the Hertz model,^[^ ^4^ ^]^ and then calculate *J* registered with each CAFM tip. However, even in such cases comparisons might be still meaningless if *R_tip_* degrades during the experiments^[^ ^23^ ^]^—two CAFM tips always degrade at different speeds because that depends on the experiments carried out, which have an intrinsic variability. Hence, for any CAFM study, it is always recommended to measure all the samples with the same tip. This was extremely complex in the past due to the fast wearing of the metal‐coated Si CAFM tips, but the current limitation system integrated into the Bruker Dimension Icon CAFM (adjusted at 110 pA in this study) makes for the first time such extremely accurate characterization possible.^[^ ^23^ ^]^ In particular, even when using standard Pt‐coated Si tips (CONTV‐PT) we are able to measure more than 3,950 *I*–*V*s without observing any degradation—this is demonstrated by repeating the first experiment at the end and observing the same result. Table S1 (Supporting Information) summarizes the experiments carried out with each CAFM tip used in this study.

## 3 Ultra‐Thin SiO_2_/n^++^Si Samples

We start by investigating the effect of RH in CAFM experiments conducted on the ultra‐thin SiO_2_/n^++^Si samples, featuring SiO_2_ thicknesses of 1.5, 2.3, 3.4, and 5.6 nm. These samples have been fabricated via thermal oxidation on highly conductive n‐type (100) Si at the laboratories of Infineon Technologies, benefiting from a fabrication process optimized over the past 30 years that enables precise control of sample thickness at atomic resolution and featuring state‐of‐the‐art quality. This is confirmed via cross‐sectional transmission electron microscopy (TEM) analyses, as shown in Figure S2 (Supporting Information). Using this type of SiO_2_/n^++^Si samples is very important because this has been the reference insulator in microelectronics over the past 70 years, and many of its properties affecting the tunneling current are well understood, including band gap (9.1 eV),^[^ ^27^ ^]^ electron effective mass (0.42 m),^[^ ^28^ ^]^ and wetting properties (hydrophilic, water contact angle ≈20.7°),^[^ ^29^ ^]^ among others. Each SiO_2_/n^++^Si wafer is cut into multiple 1.5 cm × 1.5 cm pieces and cleaned as detailed in the Methods section. Then, one of those pieces for each SiO_2_ wafer is glued to the CAFM sample holder using silver paint, and a CAFM tip is approached to its surface.

### 3.1 Determination of the Suitable Deflection Setpoint

First, we characterize the 3.4 nm SiO_2_/n^++^Si sample by applying RVS at 100 locations in air ambient (RH = 54%) with an RMN25PT300B tip. When measuring in air ambient, the presence of a water meniscus surrounding the tip/sample junction is unavoidable;^[^ ^30^, ^31^ ^]^ however, one should always ensure that the metallic tip is touching the surface of the sample and avoid the presence of water molecules between them (i.e., right below the tip), otherwise, the structure under test is not the one intended to be measured. To do so, one has to apply an *F_c_* that is high enough to penetrate the water layer at the tip/sample junction, but without indenting the sample. Ref. [32] estimated the correct *F_c_* in a CAFM experiment (using a solid Pt tip and a mechanically exfoliated 3‐nm‐thick h‐BN flake) by applying one *I–* *V* curve under *F_c_* of 1, 2, 3, 4, and 5 nN; the authors observed that *F_c_* values between 2 and 5 nN resulted in similar *I–* *V* curves, while the one at *F_c_* = 1 nN showed slightly lower currents. Hence, they concluded that the lower currents at *F_c_* = 1 nN were related to a bad tip/sample contact (presence of water molecules between the CAFM tip and the sample) and that any *F_c_* value between 2 and 5 nN was fine for their experiments. However, the differences between the *I*–*V* curves when changing *F_c_* were as small as the point‐to‐point variability in such samples, which can lead to misleading interpretations; to extract a more solid conclusion, the experiment should has been conducted statistically at multiple locations of the sample. In this CAFM study, we correctly estimate the optimal measuring conditions statistically by collecting hundreds of RVS at different *F_c_*. Note that the value of *F_c_* is in fact controlled through the deflection setpoint (DS) parameter in the CAFM control panel and that the user determines the optimal measuring conditions when stable *I–* *V* curves are observed. Hence, from a practical point of view, the important value to determine in a CAFM study is DS, not *F_c_*. *F_c_* could be later calculated after measuring the real spring constant of the tip (not nominal), which according to the manufacturer is different for each CAFM tip.

**Figure 1:**
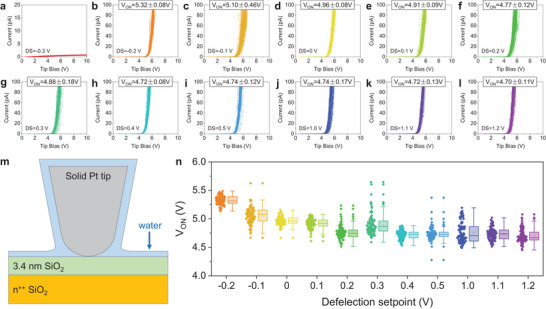
Estimating the correct deflection setpoint (DS) in a conductive atomic force microscopy (CAFM) study under relative humidity (RH) = 54%. a–l) *I*–*V* curves collected with an RMN25PT300B solid Pt tip (using a current limitation of 110 pA) on the surface of a 3.4 nm SiO_2_/n^++^Si sample, under different deflection setpoint of −0.2, −0.1, 0, 0.1, 0.2, 0.3, 0.4, 0.5, 1.0, 1.1, and 1.2 V, respectively (RH = 54%). Each plot contains 100 *I*–*V* curves collected in a 10 × 10 matrix with point‐to‐point distances of 1 µm. m) Schematic of the CAFM setup under RH = 54% when the solid Pt tip is in contact with the surface of the sample. The grey semicircle represents the solid Pt tip. The green and orange rectangles represent the sample, and the blue shape surrounding both the tip and sample represents the water film at RH = 54%. n) Statistical analysis of *V_ON_* versus deflection setpoint extracted from (b–l).

We characterize the 3.4 nm SiO_2_/n^++^Si sample at RH = 54% and under various values of DS equal to −0.3, −0.2, −0.1, 0, 0.1, 0.2, 0.3, 0.4, 0.5, 1, 1.1, and 1.2 V—first the CAFM tip is engaged under DS = 0.2 V, and then this is adjusted to each value. For each DS, we apply RVS at 100 random (fresh) positions of the sample using a current limitation of 110 pA. As **Figure** **1**a shows, using DS = −0.3 V results in no currents flowing across the tip/sample junction, indicating that the Pt tip is not touching the surface of the SiO_2_ sample. For all the other values of DS, current can be detected. When using DS = −0.2 V, the value of *V_ON_* detected is the highest, and as DS increases, the value of *V_ON_* reduces progressively, until DS = 0.2 V, above which *V_ON_* remains nearly constant (see Figure 1n). Hence, we determined that the most suitable DS to measure the 3.4 nm SiO_2_/n^++^Si samples using the RMN25PT300B tips under RH = 54% is 0.2 V. We also conducted topographic maps after the *I*–*V* curves and did not detect any signal of tip indentation into the sample for DS = 0.2 V (see Figure S3, Supporting Information).

**Figure 2:**
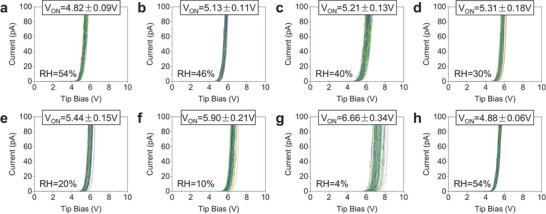
CAFM characterization of SiO_2_/n^++^ Si samples. a–g) *I–* *V* curves were collected under seven RH levels with a current limitation of 110 pA on the surface of a 3.4‐nm‐thick SiO_2_/n^++^ Si sample. Each *I*–*V* plot contains 100 *I*–*V* curves, and each is collected at a different location. h) The last group of *I–* *V* curves collected under RH = 54% (air), which is after the measurement of (g).

Next, we measure the 3.4 nm SiO_2_/n^++^Si sample at DS = 0.2 V and under RH values of 54, 46, 40, 30, 20, 10, and 4%, and we clearly observe that the mean and standard deviation of *V_ON_* is higher at lower RH, as shown in **Figure** **2**a–g. After these 2,100 *I*–*V* curves, we collected another 100 *I*–*V* curves under RH = 54% (see Figure 2h and Experimental Section) and compared them to the very first group (Figure 2a); the similarity of the data confirms that the CAFM tip integrity has been maintained. The trend observed clearly indicates that at lower RH the CAFM tip collects less current, leading to higher values of *V_ON_*. To be sure that this trend is reproducible, we repeated all these experiments two times more at different locations of the 3.4 nm SiO_2_/n^++^Si sample and obtained very similar results (see Figure S4, Supporting Information).

**Figure 3:**
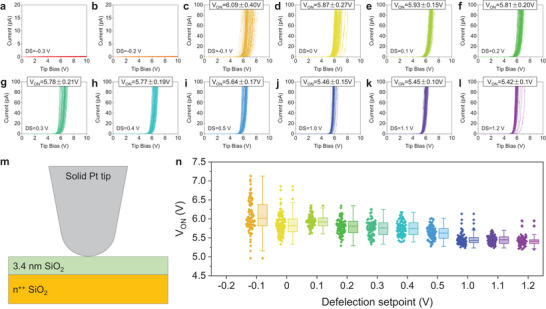
Estimating the correct DS in a CAFM study under RH = 4%. a–l) *I–* *V* curves collected with an RMN25PT300B solid Pt tip (using a current limitation of 110 pA) on the surface of a 3.4 nm SiO_2_/n^++^Si sample, under different deflection setpoint of −0.2, −0.1, 0, 0.1, 0.2, 0.3, 0.4, 0.5, 1.0, 1.1, and 1.2 V, respectively (RH = 4%). Each plot contains 100 *I*–*V* curves collected in a 10 × 10 matrix with point‐to‐point distances of 1 µm. m) Schematic of the CAFM setup under RH = 4% when the solid Pt tip is in contact with the surface of the sample. The grey semicircle represents the solid Pt tip. The green and orange rectangles represent the sample. n) Statistical analysis of *V_ON_* versus deflection setpoint extracted from (b–l).

The increase of *V_ON_* observed at lower RH levels in Figure 2 is suspiciously high. For this reason, we question ourselves whether the value of DS = 0.2 V (determined at RH = 54%) is suitable for measuring under other values of RH. We repeat the experiments shown in Figure 1 (aimed to assess the suitable value of DS) but at RH = 4%. The results are presented in **Figure** **3**. At RH = 4%, the lowest DS (−0.1, 0, and 0.1 V) show values of *V_ON_* much higher and dispersed than the rest, and they start to stabilize at around DS = 0.2 V. Hence, this indicates that (for this specific CAFM tip and sample) the suitable value of DS for measuring at RH = 4% is also 0.2 V (same than at RH = 54%), and that the *V_ON_* increase observed in Figure 2 is not related to tip/sample contact issues. It is worth noting that the value of *V_ON_* at RH = 4% with DS = 0.2 V shown in Figure 3f (5.8 ± 0.2 V) is lower than the one measured under identical conditions in Figure 2g (6.6 ± 0.3 V); the reason is that Figures 2 and 3 have been collected using different RMN25PT300B tips (see Table S1, Supporting Information).

More importantly, the values of *V_ON_* collected under different DS values at RH = 4% (Figure 3n) are always significantly higher than those collected at RH = 54% (Figure 1n). In other words, at RH = 4%, one cannot get as high currents as detected at RH = 54%, no matter how much he/she pushes with the CAFM tip (that is, no matter how high the value of DS, or *F_c_*, is). This observation can only be related to the reduction of the size of the water meniscus at RH = 4%,^[^ ^25^ ^]^ as this is the only parameter changed in the setup, and it indicates that the water meniscus surrounding the tip/sample junction is driving current (and reducing *V_ON_*). While pure water is typically insulating, the thin water layer on the surface of the samples analyzed is typically conductive due to the presence of ions,^[^ ^33^ ^]^ and it has been shown that it contributes to a spreading of the electrical field on the surface of the sample.^[^ ^31^ ^]^ Hence, in Figure 2a (at RH = 54%) the value of *A_eff_* is higher than in Figure 2g (at RH = 4%), and therefore more current is detected and *V_ON_* is lower. This observation is in line with previous studies in capacitors using ultra‐thin SiO_2_ as dielectric: a smaller device size results in lower leakage currents (or higher *V_ON_*) and higher device‐to‐device variability.^[^ ^34^ ^]^ This concept is schematically depicted in Figure S5a,b (Supporting Information).

To be completely sure that this trend is reproducible, we repeat all these experiments using CONTV‐PT tips under DS = 0.8 V, which is optimal for this type of tip and sample. We observed that the results are similar (see Figures S6 and S7, Supporting Information). After the 1,200 *I*–*V*s and 300 *I*–*V*s under RH = 54 and 4%, respectively (12 DSs under each RH, with 100 *I*–*V*s at each DS under RH = 54%; and 12 DSs under each RH, with 25 *I*–*V*s at each DS under RH = 4%), we apply RVS at another fresh area with DS = 0.8 V to check the conductivity of the tip and to ensure that the changes of *V_ON_* we observed with different DSs are not related to tip degradation (see Figure S8, Supporting Information). In that case, the values of *V_ON_* observed are always higher than those obtained with the RMN25PT300B tips, which could be because of the lower spring constant of these tips (0.2 N m^−1^ versus 18 N m^−1^), which for the same DS leads to a lower *F_c_*. But the trend is identical, at both RH (54% and 4%) the currents increase at higher DS, but the values of *V_ON_* detected at RH = 4% are always significantly higher than those detected at RH = 54%. It should be highlighted that, for this type of CAFM tip (CONTV‐PT), the suitable DS depends on the RH, being 0.4 V the most suitable at RH = 54% and 0.7 V for RH = 4% (see Figures S6 and S7, Supporting Information). The reason may be that the attractive *F_c_* introduced by the water meniscus at RH = 54% is much weaker at RH = 4%, and hence one has to apply higher force (increase DS) in order to get a similar tip/sample contact (and stable electrical data). This is an extremely important observation (affecting all subsequent experiments) that has been overlooked in all previous CAFM studies analyzing the effect of RH with soft Pt‐coated Si tips.^[^ ^20^, ^21^ ^]^

### 3.2 Trend Observed for Different SiO_2_ Thicknesses

**Figure 4:**
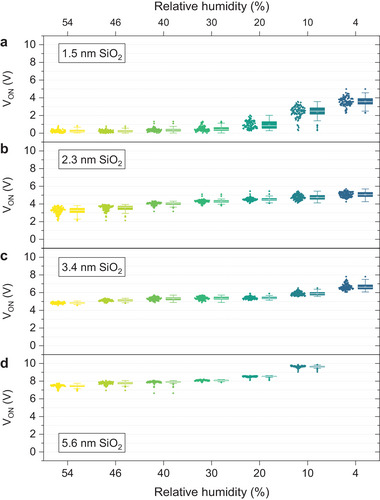
CAFM characterization of SiO_2_/n^++^ Si samples. i–l) Statistical analysis on *V_ON_* versus RH levels, for 1.5, 2.3, 3.4, and 5.6 nm, respectively. Each box in the statistical analysis plots includes 100 data points. Inside each box, a thick solid white line indicates the median value of the *V_ON_* calculated from the 100 *I*–*V* curves, a white dot indicates the mean value of *V_ON_*, while the error bar represents the standard deviation of the *V_ON_* calculated from the same 100 data points.

We collect another 2,200 *I*–*V* curves for each one of the other SiO_2_ samples available, with thicknesses of 2.3, 3.4, and 5.6 nm (see Figures S9‐S11, Supporting Information), at DS = 0.2 V and RH values of 54, 46, 40, 30, 20, 10, and 4%. In all cases, the conductivity of the CAFM tip used for the experiments was preserved (see Figure S12, Supporting Information). The statistical analyses of *V_ON_* (see **Figure** **4**) clearly indicate that (for a given RH) *V_ON_* is higher in thicker samples and that within each sample the same trend is repeated: the mean and standard deviation of *V_ON_* increase at lower RH levels. It is noteworthy that the data box for the 5.6‐nm‐thick sample under RH = 4% is missing; the reason is that the CAFM can only apply voltages within the range of −10 V to +10 V, which is insufficient for the observation of current above the noise level (for a thick sample at that RH).

### 3.3 Trend Observed via Current Maps

**Figure 5:**
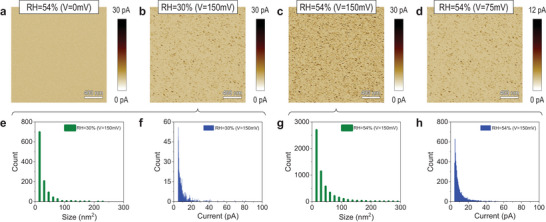
CAFM characterization of 1.5‐nm‐thick SiO_2_/n^++^ Si sample. a–d) CAFM current maps were collected at four different areas, under different RH levels, and with different voltages. a) CAFM current map collected under RH = 54% without applying voltage. b) CAFM current map collected under RH = 30%, with a voltage of 150 mV (V1). c) CAFM current maps collected under RH = 54%, with a voltage of 150 mV (V1). d) CAFM current maps collected under RH = 54%, with a voltage of 75 mV (V2), e,f) Statistical analysis of current spots size (area) and the current value of the conductive spots detected in (b) respectively. g,h) Statistical analysis of current spot size (area) and current value of the conductive spots detected in (c) respectively.

Next, we explore if this trend is also observed from CAFM current maps collected in contact mode. First, we measure the 1.5 nm SiO_2_ sample under RH = 30% without applying any voltage, which shows no current (see **Figure** **5**a). Then, we increase the voltage until starting to detect current above the noise level, which is achieved at 150 mV. This voltage can be considered as the estimation of *V_ON_* from current maps, and it is normally different than the one estimated from *I–* *V* plots because the number of locations tested is much higher (65,536 in current maps versus 512 in *I–* *V* plots), and hence, the probability of finding defective (electrically weaker) locations is higher. At 150 mV, the images collected show tiny current spots randomly distributed along the surface of the sample (Figure 5b), which correspond to electrically weakest locations due to the presence of atomic defects (dangling bonds, impurities) and/or local SiO_2_ thickness reductions. Figure 5b looks like the typical CAFM current map obtained when measuring ultra‐thin SiO_2_, and it has been also observed in many other studies.^[^ ^11^, ^35^ ^]^ By using the CAFM software we determine that the density of current spots driving maximum currents above 5 pA is 276.5 µm^−1^ and that their size and maximum currents driven are 28.64 ± 31.10 nm^2^ and 9.26 ± 7.21 pA, respectively (see Figure 5e,f).

Next, we measure the same sample (at a different fresh location) under RH = 54% by applying the same voltage (150 mV), and we clearly observe (see Figure 5c) that the density of current spots registered is higher (1,394.25 µm^−1^), and also that their size and maximum currents driven are higher (41.60 ± 48.67 nm^2^ and 12.62 ± 18.41 pA, respectively, see Figure 5g,h). These observations indirectly support the conclusions extracted from Figure 2: the lower *V_ON_* is equivalent to the observation of more and larger current spots driving higher currents at a given voltage. We additionally estimate the value of *V_ON_* from current maps under RH = 54% by applying the minimum voltage needed to observe current above the noise level, and we obtain a value of 75 mV (see Figure 5d). This observation directly supports the conclusions extracted from Figure 2. To be completely sure that this trend is reproducible, we repeate the experiments in another two locations of the sample, and identical conclusions could be extracted (see Figure S13, Supporting Information). Moreover, the flat topography registered during all the current maps is very similar to that obtained in tapping mode without applying any bias (see Figure S14, Supporting Information), which strongly supports the good tip/sample contact during the scans and the validity of the trend observed.

### 3.4 Quantifying the Effect of the Water Meniscus

By looking at the measurements, it is possible to estimate how many times the currents measured at RH = 54% are higher than those measured at RH = 4%. It can be calculated that this factor ranges between 100 and 1,000, depending on the sample and DS used (see Figure S15, Supporting Information). Considering, for simplicity, a negligible water meniscus at RH = 4%, the value of *A_eff_* when measuring the insulating SiO_2_/n^++^Si sample at RH = 4% equals to *A_c_*, which in our setup (according to the Hertz model and SEM images of *R_tip_*) is roughly 35 nm^2^ (see Note S1, Supporting Information). The current measured by the CAFM tip under RH = 54% (with water meniscus surrounding the tip/sample junction) can be expressed as *I*_54%_ = *A_c_* × *J* + *A_w_* × *J_w_*, where *A_w_* is the area with tip/water/insulator structure across which electrons can flow, and *J_w_* is the average current density across *A_w_*. The problem is that calculating *A_w_* and *J_w_* is extremely challenging. *A_w_* depends on the thickness of the water film on the surface of both tip and sample, which depends not only on the RH, but also on the wetting properties of the surface of both tip and sample. Ref. [31] simulated the electrical field spreading the junction of a Pt‐coated Si tip landed on the surface of a SiO_2_ sample by considering water layer thicknesses of 1 and 3 nm, but it did not specify how the thickness of the water layer was obtained. Reference ^[^ ^30^ ^]^ measured the size of the water meniscus formed between a SiN tip and a Si substrate using an environmental SEM and observed that its radius ranges between ≈100 nm at RH = 40% and ≈400 nm at RH = 60%. However, the width of the water meniscus in the SEM might be different than on the CAFM sample holder. So far, there is no widespread method to calculate the thickness of the water films on the surfaces of the tip and sample, and also no clear way to measure accurately the width of the water meniscus. Calculating *J_w_* is also very complex because the biased structure within *A_w_* is Pt/water/SiO_2_, but the thickness of the water layer is highly inhomogeneous; moreover, the conductivity of the water film on the surface of the sample is unknown and there is no widespread method to measure it (not to mention in situ, which is how such measurement should be done). For simplicity, the currents measured under RH = 54% can also be described as *I*_54%_ = *A_eff._*_54%_ × *J*, meaning that the increase of current produced by the water meniscus can be understood as an increase of *A_eff_*. Therefore, it can be concluded that measuring under RH = 54% increases the value of *A_eff_* (now, *A_eff._*_54%_) in a factor ranging from 100 to 1,000, which is the same as saying that *A_eff.54%_* ranges between 3,500 and 35,000 nm^2^, or that the radius of *A_eff.54%_* (namely *r_eff.54%_*) ranges between 33 and 106 nm. Interestingly enough, the value of *r_eff.54%_* obtained in this study by CAFM electrical measurements at RH = 54% is very similar to the water meniscus radius observed via environmental SEM at RH = 40% in ref. [30] (≈100 nm).

However, it should be emphasized that, when trying to quantify the current increase produced by the presence of the water meniscus, an important dilemma arises. Some scientists may think that one should compare the currents registered at different *RHs* using the same DS, which leads to the same *F_c_*. However, some other scientists may think that the data should be compared when using the optimal DS for each RH. In the case of our RMN25PT300B tips (*k* = 18 N m^−1^), the suitable value of DS to measure at RH = 54% and RH = 4% is the same (0.2 V, see Figures 1 and 3); hence, for this type of tips there is no problem and all the measurements can be easily compared. However, in the case of our CONTV‐PT tips (*k* = 0.2 N m^−1^) the suitable value of DS depends on RH: 0.4 V at RH = 54% and 0.7 V at RH = 4% (see Figures S4 and S5, Supporting Information). Therefore, making direct comparisons of currents at different RH could be a bit more difficult when using soft CAFM tips.

## 4 Mechanically Exfoliated h‐BN Sample

Next, we try to find out whether this trend (higher currents at higher RH) is also observable in other insulating samples with different surface properties. To do so, we select a sample consisting of mechanically exfoliated multilayer h‐BN, which is a 2D layered material with a band gap of ≈5.9 eV;^[^ ^12^ ^]^ its surface is hydrophobic (water contact angle: ≈79°),^[^ ^36^ ^]^ meaning that it is expected to accumulate less water at the tip/sample junction than the (hydrophilic) SiO_2_/n^++^Si samples. When analyzing this sample via CAFM we use a new CONTV‐PT tip. The reason is that the RMN25PT300B solid Pt tips used in this study occasionally damaged the ultra‐thin mechanically exfoliated micro‐flakes while scanning at the edge of the flakes, due to their high spring constant (*k* = 18 N m^−1^), which easily results in relatively high *F_c_* even when applying a low deflection setpoint in the CAFM configuration panel. In particular, the damage consisted of the detachment of the h‐BN flakes from the Ru substrate, because their adhesion by van der Waals forces is weaker than other types of chemical bonds. These problems were never observed when using CONTV‐PT tips (*k* = 0.2 N m^−1^).

**Figure 6:**
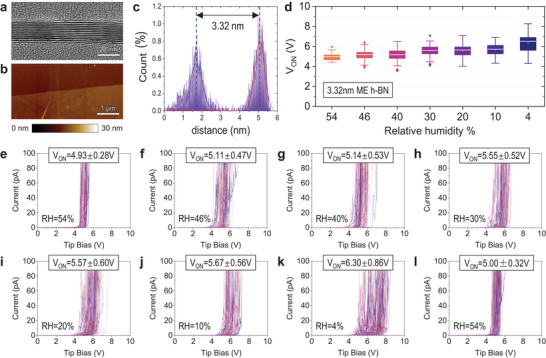
CAFM characterization of mechanically exfoliated h‐BN/Ru sample. a) Cross‐sectional TEM image of a ≈3.32 nm mechanically exfoliated h‐BN. b) AFM topography map collected at the edge of the target h‐BN flake. c) Thickness analysis of the exfoliated h‐BN flake in (b). d) Statistical analysis on *V_ON_* versus RH levels, for mechanically exfoliated ≈3.32 nm h‐BN. Each box in the statistical analysis plots includes 100 data points. Inside each box, a thick solid white line indicates the median value of the *V_ON_* calculated from the 100 *I*–*V* curves, a white dot indicates the mean value of *V_ON_*, while the error bar represents the standard deviation of the *V_ON_* calculated from the same 100 data points. e–k) *I*–*V* curves were collected under seven RH levels with a current limitation of 110 pA on the surface of the ≈3.32‐nm‐thick h‐BN/Ru sample. Each *I*–*V* plot contains 100 *I*–*V* curves, and each is collected at a different location. l) The last group of *I–* *V* curves collected under RH = 54% (air), which is after the measurement of (k).

We mechanically exfoliate several h‐BN flakes and transfer them onto a flat Ru substrate (see Experimental Section), whose root mean square (RMS) surface roughness is 310 pm and leads to a tip/sample (i.e., Pt/Ru) contact resistance of 500 KΩ.^[^ ^37^ ^]^ This type of substrate is highly recommendable for the CAFM characterization of 2D materials because it avoids the formation of interfacial gaps,^[^ ^37^ ^]^ which would result in artificially insulating locations. Note that the only gaps possible between the h‐BN and the Ru substrate are those related to wrinkles, but we intentionally excluded such regions from our analysis using the CAFM topographic map. The exfoliated h‐BN is chosen as a reference sample for studying the RH effect on h‐BN samples because this method produces the highest‐quality 2D material with relatively low interlayer defects.^[^ ^38^, ^39^ ^]^ We then scan the edge of the target h‐BN flakes by using the AFM with a Si tip under tapping mode (see **Figure** **6**a) and measure its thickness through histogram analysis (see Figure 6c), revealing a value of 3.32 nm. We also analyze the morphology of the sample via cross‐sectional TEM, which reveals an excellent layered structure without atomic defects (see Figure 6b and Figure S16a, Supporting Information).

Subsequently, the same measurement protocol as for the SiO_2_ samples is replicated for the mechanically exfoliated h‐BN, where 100 *I*–*V* curves are collected under each RH level (54, 46, 40, 30, 20, 10, and 4%), using a current limitation of 110 pA (see Figure 6e–k). After that, we also collect an additional set of 100 *I*–*V* curves under RH = 54% after that to confirm that the CAFM tip remained in a reliable condition (see Figure 6l; Figure S17, Supporting Information). The trend observed in this mechanically exfoliated h‐BN flake on Ru is very similar to that observed in the SiO_2_/n^++^Si samples: at lower RH the sample drives less and more inhomogeneous current, which can also be seen by an increase of the mean and standard deviation of *V_ON_*. Specifically, the *V_ON_* changed from 4.93 ± 0.28 V under RH = 54% to 6.30 ± 0.86 V under RH = 4%.

Note that the CAFM experiments on the SiO_2_/n^++^Si samples have been conducted using an RMN25PT300B tip (*k* = 18 N m^−1^), while those in the mechanically exfoliated h‐BN sample have been conducted using a CONTV‐PT tip (*k* = 0.2 N m^−1^). This indicates that the dependence of the current with the RH observed in Figures 2 and 4 cannot be related to unwanted phenomena like tip indentation (when using the stiffer tip) or interfacial water molecules at the tip/sample junction (when using the milder tip).

## 5 CVD‐Grown h‐BN Samples

We also measure the electrical properties of h‐BN produced by CVD; these samples have attracted the interest of the semiconductor industry because the CVD method allows synthesizing the 2D materials at the wafer scale, which enables the fabrication of electronic circuits and microchips.^[^ ^40^ ^]^ However, CVD‐grown 2D materials are known to contain higher densities of defects, including atomic vacancies, lattice distortions, and impurities.^[^ ^37^, ^38^, ^41^ ^]^ Hence, it is interesting to analyze if the density of defects in the material has any effect on the dependence of *V_ON_* with respect to RH.

**Figure 7:**
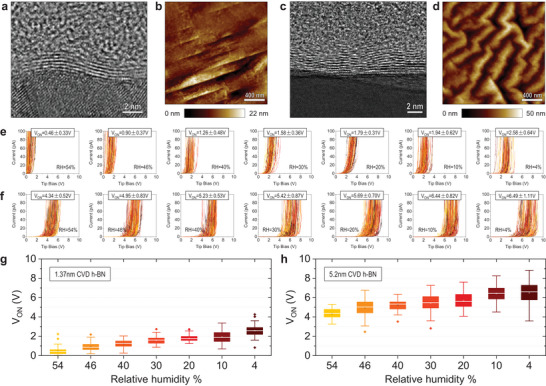
CAFM characterization of CVD‐grown multilayer h‐BN/Cu samples. a,b) Cross‐sectional TEM image and AFM topography map of CVD h‐BN sample 1: ≈1.37 nm h‐BN/Cu, respectively. c,d) Cross‐sectional TEM image, and AFM topography map of CVD h‐BN sample 2: ≈5.2 nm h‐BN/Cu, respectively. e) *I*–*V* curves collected under seven RH levels with a current limitation of 110 pA on the surface of a CVD‐grown ≈1.37‐nm‐thick h‐BN/Cu sample. Each *I*–*V* plot contains 100 *I*–*V* curves, and each is collected at a different location. f) *I*–*V* curves collected under seven RH levels with a current limitation of 110 pA on the surface of a CVD‐grown ≈5.2‐nm‐thick h‐BN/Cu sample. Each *I*–*V* plot contains 100 *I*–*V* curves, and each is collected at a different location. g,h) Statistical analysis on *V_ON_* versus RH levels, for CVD‐grown ≈1.37 and ≈5.2 nm h‐BN, respectively. Each box in the statistical analysis plots includes 100 data points. Inside each box, a thick solid white line indicates the median value of the *V_ON_* calculated from the 100 *I*–*V* curves, a white dot indicates the mean value of *V_ON_*, while the error bar represents the standard deviation of the *V_ON_* calculated from the same 100 data points.

We conduct CAFM measurements under different RH levels on two commercial multilayer h‐BN samples grown by the CVD method on Cu foils. The thicknesses of these two CVD‐grown h‐BN samples measured via high‐resolution cross‐sectional TEM are ≈1.3 and 5.2 nm, respectively (see **Figure** **7**a,c). The images also reveal a higher density of defects compared to mechanically exfoliated samples, as expected^[^ ^37^, ^38^, ^41^ ^]^ (see Figure S16b,c, Supporting Information). The AFM topography maps, obtained in tapping mode, are presented in Figures 7b,d; the relatively high surface roughness observed is related to the Cu substrate underneath the h‐BN. However, this is not a concern for the CAFM measurements because the h‐BN is perfectly attached to the Cu foil; as it is directly grown (not transferred) the formation of interfacial gaps is extremely unlikely. In these CVD‐grown multilayer h‐BN samples we rarely found wrinkles, probably because the relatively high surface roughness of the Cu foil relaxes the compressive strain produced during the cooling down process.^[^ ^42^ ^]^ Moreover, the surface roughness of the Cu foil in this commercial multilayer h‐BN/Cu samples is higher than in other studies growing monolayer h‐BN/Cu probably due to the longer growth time.^[^ ^37^ ^]^

By employing the same measurement protocol as described earlier, we collect 100 *I*–*V* curves under each RH level (54, 46, 40, 30, 20, 10, and 4%, see Figure 7e,f), followed by an additional set of 100 *I*–*V* curves collected under RH = 54% (see Figure S18, Supporting Information). As these samples are continuous films, we use an RMN25PT300B solid Pt tip. Statistical analyses of *V_ON_* under different RH levels again show the same trend as in SiO_2_ and mechanically exfoliated h‐BN: at lower RH levels the samples look more insulating and inhomogeneous (see Figure 7g,h). The *V_ON_* of a CVD‐grown 1.37 nm h‐BN changed from 0.46 ± 0.33 V under RH = 54% to 2.58 ± 0.64 V under RH = 4%, and the *V_ON_* of a CVD‐grown 5.2 nm h‐BN changed from 4.34 ± 0.52 V under RH = 54% to 6.49 ± 1.11 V under RH = 4%.

## 6 Mechanically Exfoliated MoS_2_ Samples

**Figure 8:**
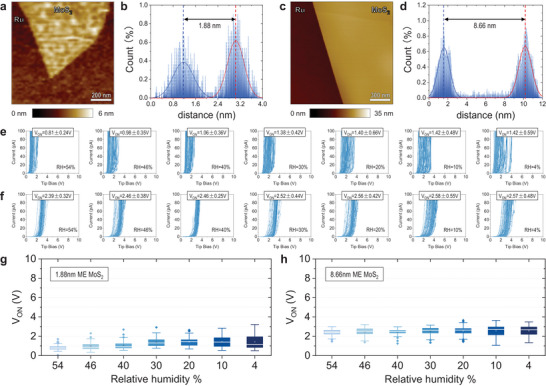
CAFM characterization of mechanically exfoliated MoS_2_/Ru samples. a,b) AFM topography map and thickness analysis histogram of mechanically exfoliated MoS_2_ flake 1: ≈1.88 nm MoS_2_/Ru, respectively. c,d) AFM topography map and thickness analysis histogram of mechanically exfoliated MoS_2_ flake 1: ≈8.66 nm MoS_2_/Ru, respectively e) *I*–*V* curves collected under seven RH levels with a current limitation of 110 pA on the surface of a mechanically exfoliated ≈1.88 nm MoS_2_ sample. Each *I*–*V* plot contains 100 *I*–*V* curves, and each is collected at a different location. f) *I–* *V* curves collected under seven RH levels with a current limitation of 110 pA on the surface of a mechanically exfoliated ≈8.66 nm MoS_2_ sample. Each *I*–*V* plot contains 100 *I*–*V* curves, and each is collected at a different location. g,h) Statistical analysis on *V_ON_* versus RH levels, for mechanically exfoliated ≈1.88 and ≈8.66 nm MoS_2_, respectively. Each box in the statistical analysis plots includes 100 data points. Inside each box, a thick solid white line indicates the median value of the *V_ON_* calculated from the 100 *I*–*V* curves, a white dot indicates the mean value of *V_ON_*, while the error bar represents the standard deviation of the *V_ON_* calculated from the same 100 data points.

Next, we analyze the effect of RH in CAFM measurements of 2D semiconductors. MoS_2_ is also a layered material, with a band gap of 1.2 eV^[^ ^43^ ^]^ and a hydrophobic surface (water contact angle ≈89°),^[^ ^44^ ^]^ which we selected for study here because it has become very popular in the community of 2D materials and beyond silicon electronics. We mechanically exfoliated several MoS_2_ flakes on Ru substrates (see Experimental section) and measured the thickness of the flakes by scanning the edge of each flake under AFM in tapping mode (see **Figure** **8**a,c). Analysis of the histograms revealed thicknesses of 1.88 and 8.66 nm for the two exfoliated MoS_2_ flakes (see Figure 8b,d). We follow the same measuring protocol as for the other materials, collecting 100 *I*–*V* curves under each RH level for each MoS_2_ flake (54, 46, 40, 30, 20, 10, and 4%, see Figure 8e,f), followed by an additional set of 100 *I–* *V* curves collected under RH = 54% for each flake (see Figure S19, Supporting Information).

Similar to all other insulating samples, the currents measured on the 1.88 nm MoS_2_ sample seem to be significantly affected by the RH: *V_ON_* was 0.81 ± 0.24 V under RH = 54% and 1.42 ± 0.59 V under RH = 4% (see Figure 8g). However, the effect of the RH seems to be negligible in the 8.66 nm MoS_2_ sample: *V_ON_* was 2.39 ± 0.32 V under RH = 54% and 2.57 ± 0.48 V under RH = 4% (see Figure 8h). The reason behind this observation is that the lateral conductivity of the semiconducting MoS_2_ samples (that is, the lateral electron mean free path) increases with the thickness. This has been proved in other semiconducting materials (such as Si)^[^ ^45^ ^]^ and even water.^[^ ^31^ ^]^ The electron mean free path in monolayer CVD‐grown MoS_2_ has been estimated to be between 1 and 3 nm,^[^ ^46^ ^]^ and in few‐layers MoS_2_ it has been estimated to be between 27 and 40 nm.^[^ ^47^ ^]^ In our 8.66 nm MoS_2_ sample we can safely assume that the electron mean free path will be slightly higher because it is significantly thicker. Assuming (quite reasonable) values of the electron mean free path between 60 to 100 nm, the values of *A_eff_* for the 8.66 nm MoS_2_ sample would range between 11,300 and 31,400 nm^2^—the electron mean free path equals to *r_eff_*. In other words, the increase of *A_eff_* produced by the intrinsically high lateral conductivity of the semiconducting MoS_2_ is comparable to the increase of *A_eff_* produced by the water meniscus, and therefore it masks the effect of RH (see Figure 8h). This concept is schematically depicted in Figure S4c,d (Supporting Information). On the contrary, in the 1.88 nm MoS_2_ sample the electron mean free path is lower than in the 8.66 nm MoS_2_ sample (and it does not increase *A_eff_* significantly), making visible the effect of the RH (*A_eff_* increase due to the water meniscus, similar to the insulating samples). This concept is schematically depicted in Figure S4e,f (Supporting Information). Making the same type of calculations as for SiO_2_ and h‐BN, it can be concluded that the value of *A_eff._*_54%_ for the 1.88 nm MoS_2_ sample ranges between 106 and 17,800 nm^2^.

## 7 Metallic Samples

**Figure 9:**
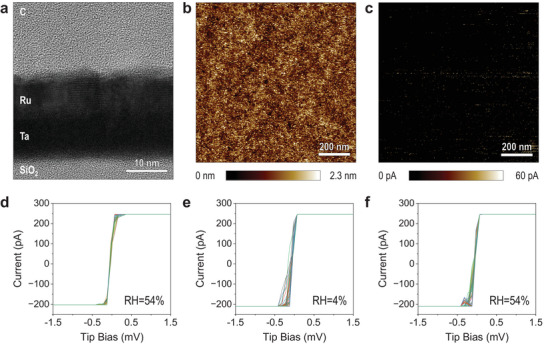
CAFM characterization of metallic (Ru) sample. a) Cross‐sectional TEM image of a Ru/Ta/SiO_2_/Si sample. b,c) CAFM topography map and current map collected on the surface of the Ru/Ta/SiO_2_/Si without applying bias, under RH = 54%, respectively. d–f) *I–* *V* curves were collected under two RH levels with a current limitation of −210, +250 pA on the surface of a Ru/Ta/SiO_2_/Si sample. Each *I*–*V* plot contains 100 *I*–*V* curves, and each is collected at a different location.

Finally, we analyze what is the effect of the RH when measuring metallic samples. **Figure** **9**a shows the cross‐sectional TEM image of the sample used, which consists of a 20‐nm‐thick bilayer stack made of Ru/Ta on a standard 300 nm SiO_2_/Si wafer. The surface of this sample is very flat, and its RMS roughness measured via topographic AFM maps collected in tapping mode is only 310 pm (see Figure 9b). When a current map is collected by applying a constant voltage of only 1 mV all the regions of the sample appear to be highly conductive (see Figure 9c).

Next, we apply RVS on the surface of the Ru/Ta/SiO_2_/Si sample with 20,480 points per *I–* *V*. The surface of the Ru is grounded and the RVS is applied to the CAFM tip, meaning that the current only flows along the Ru film. The SiO_2_/Si serves as a substrate and the 10 nm interfacial Ta film acts as an adhesion layer. In this case, we did not use the 110‐pA current limitation because the contact resistance is much lower, which produces a limited amount of data points in the *I–* *V* curves—and inaccurate resistance calculation. First, we collect 100 *I*–*V* curves at random locations under RH = 54%, then under RH = 4%, and then under RH = 54% (again), and we compare all of them. As in this experiment, no current limitation is used, and the RVS is not interrupted, which results in a central part in which the currents increase linearly at both polarities before they saturate at current levels of −200 and 250 pA. The three *I*–*V* plots (Figure 9d–f) show very similar results, with all the *I–* *V* curves nearly overlapping. The variability from one tested location to another is negligible, and there is no dependence with the RH. Figure 9f confirms that the CAFM tip integrity is maintained during the entire set of experiments.

In the case of the metallic sample, there is not much to discuss because the *I*–*V* plots look identical under RH = 54% and RH = 4%. In fact, the CAFM is not the most appropriate technique to analyze the electronic properties of conductive materials; scanning tunneling microscopy may be more suitable to analyze imperfections with nanoscale and even atomic resolution. Nevertheless, it is important to understand the surface roughness and contact resistance of metallic samples when being used as substrates of insulating and semiconducting materials in CAFM studies.

## 8 Discussion

In previous studies, the presence of the water meniscus at the tip/sample junction has been demonized. Refs. [19, 20] claimed that when measuring ultra‐thin insulating materials, CAFM measurements in air ambient led to lower currents than in vacuum due to the presence of contaminants at the tip/sample junction. However, such a claim indicates that the measurement was not done correctly. As we have demonstrated, the water meniscus surrounding the tip/sample junction produces an increase of current. The reason why the authors from refs. [19, 20] detected less current in air ambient should be the presence of an ultra‐thin layer of water between the tip and the sample (i.e., right below the tip), which reduces the current because it adds one additional barrier for electron tunneling. This may have happened because the contact force may not have been high enough to ensure a good tip/sample contact. This behavior was also observed in ref. [21] which, by combining CAFM data with computational analysis using Ginestra software, determined that the current reduction was related to the presence of a 1‐nm‐thick interfacial water layer between the apex of a Pt‐coated Si tip and the surface of a TiO_2_/SiO_2_ sample. Other studies have also observed artificially low currents in CAFM measurements conducted in air ambient due to the application of a too‐low contact force.^[^ ^32^ ^]^

Another factor that has contributed to demonize the presence of a water meniscus at the CAFM tip/sample junction is the abundant technical notes published by CAFM manufacturers, which present poor images labeled as “*in air*” (or “*in ambient*”) and excellent images labeled as “*in low humidity*” or “*in vacuum*”—with the only aim of convincing readers of buying the environmental chamber that they are selling. However, those technical notes rarely present enough data to extract solid conclusions, and they never present CAFM tip integrity tests after the measurements.

### 8.1 Understanding the Water Meniscus

Our systematic CAFM study demonstrates that the presence of the water meniscus is not necessarily bad; in fact, it can be even beneficial in some aspects. The reason is that the water meniscus helps to stabilize the tip/sample contact because it acts as an anti‐repulsive force. This can be observed by the fact that some of our measurements under RH = 4% (without water meniscus) show instabilities, that is, upward and downward trends in the same RVS (see Figure S20, Supporting Information). Moreover, in lateral scans collected in contact mode, the water meniscus acts as a lubricant, meaning that the lateral frictions are lower at higher RH,^[^ ^48^ ^]^ which helps to preserve the CAFM tip.

However, one has to be aware that when measuring insulating materials via CAFM in air ambient (i.e., with a water meniscus surrounding the tip/sample junction), the *A_eff_* being analyzed can be significantly larger than *A_c_*. At least that is what we clearly observe in spectroscopic *I*–*V* plots in which the CAFM tip is static, which show values of *A_eff_* ranging from 3,500 and 35,000 (or *r_eff_* between 33 and 106 nm). This conclusion can look counter‐intuitive because the current maps clearly show current spots with diameters smaller than ≈5 nm (see Figure 3), meaning that *A_eff_* cannot be much higher than ≈20 nm^2^. The reason is that in this case, the CAFM tip is moving, and in this dynamic tip/sample junction the effect of the water meniscus seems to be much smaller. For example, ref. [2] showed that the atomic lattice of graphene can be measured using a CAFM dotted with standard PtSi tips (*R_tip_* = 25 nm) working under RH between 20 and 50%, but only when scanning at high frequencies above 15 Hz. Hence, the lateral resolution of the CAFM (or, in other words, the value of *A_eff_* when working at RH above 20%), depends on the scanning frequency. We confirm this hypothesis by measuring current maps at different scanning frequencies (0.5 and 4 Hz) on the 1.5 nm SiO_2_/n^++^Si sample (see Figure S21, Supporting Information).

All the measurements presented in this study under RH = 54% follow the fundamental physical and electronic theories^[^ ^31^ ^]^: i) for all groups of samples, the current across thinner samples is lower (this can be seen comparing the SiO_2_ samples between them, the CVD‐grown samples between them, or the MoS_2_ samples between them); ii) the insulating samples show less current than the semiconducting samples (this can be seen by the fact that 3.32 nm mechanically exfoliated h‐BN shows less currents than 8.66 nm mechanically exfoliated MoS_2_); iii) the samples with higher densities of defects exhibit higher currents, which are more inhomogeneous (this can be seen by the fact that 3.32 nm mechanically exfoliated h‐BN shows less leakage current than 5.2 nm CVD‐grown h‐BN). These observations further indicate that CAFM working in air ambient can be reliably used to compare the electrical properties of insulating, semiconducting, and conducting materials. At the same time, we recommend making always relative comparisons between samples analyzed under identical conditions, and we are reluctant to trust most absolute values reported with CAFM working in air ambient due to the uncertainties associated with the water meniscus.

### 8.2 The Negligible Role of Sample Wetting Properties

The fact that we observe a similar trend in SiO_2_/n^++^Si and h‐BN samples (despite being the surface of the first one hydrophilic and the surface of the second one hydrophobic) indicates that (when using metallic CAFM tips) the wetting properties of the surface of the insulating sample plays no role in the amount of current detected—the water accumulates anyway at the tip/sample junction at high RH levels. It is unclear what would happen if the CAFM tip would be also hydrophobic. Boron‐doped diamond tips are sometimes used in CAFM science, although much less than the metallic ones due to their higher cost, higher radius, and high stiffness (which can damage most samples much more easily). It is known that the surface of oxygen‐terminated boron‐doped diamond films is hydrophilic (water contact angle 22°–25°), and that the surface of hydrogen‐terminated boron‐doped diamond films is hydrophobic (water contact angle 117.5°). However, to the best of our knowledge, there is no study reporting the wetting properties of commercially available CAFM probes made of boron‐doped diamond, and CAFM tips suppliers do not inform customers if the surface of their boron‐doped diamond tips is terminated in oxygen or diamond – we have asked a couple of suppliers and they do not know. Hence it is unclear whether boron‐doped diamond CAFM tips are hydrophilic or hydrophobic. We believe that the RH is the most critical parameter controlling the amount of water at the tip/sample junction. In any case, as we are utilizing the most widely used CAFM tips (metallic ones, both solid Pt and Pt‐coated Si tips) our conclusion (the wetting properties of the samples play no role) is applicable to most studies in the literature.

## 9 Conclusion

We have analyzed the effect of RH in CAFM experiments using an ultra‐reliable measuring protocol based on: i) the use of solid Pt nanoprobes, ii) the use of 110 pA current limitation, iii) analyzing more than 17,000 locations of the samples under test, iv) verification of tip conductivity after all the tests, v) measuring at seven RH levels (54, 46, 40, 30, 20, 10, and 4%), and vi) analyzing ten different samples, including insulating, semiconducting and conducting materials of different thicknesses (1.5, 2.3, 3.4, and 5.6 nm SiO_2_, 3.32 nm mechanically exfoliated h‐BN, 1.37 and 5.20 nm CVD‐grown h‐BN, 1.88 and 8.66 nm mechanically exfoliated MoS_2_, and 10 nm Ru). We clearly observe that: i) the suitable deflection setpoint to use during the measurements might change with the RH, especially when using CAFM tips with a lower spring constant (≈0.2 N m^−1^), due to the change in the anti‐repulsive *F_c_* provoked by the changes in the amount of water (i.e., size of water meniscus) at the tip/sample junction; ii) when analyzing insulating materials and ultra‐thin (below ≈5 nm) semiconducting materials (i.e., materials with zero or very small electron mean free path), measuring at higher RH levels results in the collection of higher and more homogeneous currents, which are related to the increase of the effective area across which electrons can flow at the tip/sample junction (*A_eff_*), due to the presence of a conducting water meniscus; iii) conducting materials and semiconducting materials thicker than ≈5 nm do not exhibit any dependence with the RH, probably because the higher lateral electron mean free path in this type of materials increases *A_eff_* significantly, masking the increase of *A_eff_* produced by the water meniscus; and iv) the registration of higher currents at higher RH levels is observed independently on the wetting properties of the insulating samples (i.e., for both hydrophobic and hydrophilic). This article concludes a long debate about the effect of RH in CAFM and educates the community on how to reliably utilize this technique.

## 10 Experimental Section

### Synthesis of SiO_2_/n^++^ Si Samples

Industrial‐quality SiO_2_ films were utilized with thicknesses of 1.5, 2.3, 3.4, and 5.6 nm, grown via rapid thermal oxidation on highly conductive n‐type (100) Si substrates. The utilization of highly doped Si substrates serves to nullify potential differences that would otherwise manifest on standard Si substrates. These n^++^Si substrates have a resistivity ranging from 0.0025 to 0.0035 Ω cm. This meticulous refinement yielded samples characterized by minimal impurity content, thickness uniformity, and well‐defined interfaces. Sample thickness verification was conducted employing ellipsometry (Therma‐Wave Optiprobe 3290DUV).

### Fabrication of Mechanically Exfoliated h‐BN and MoS_2_ Samples

The substrates consisted of 10 nm Ru/10 nm Ta/300 nm SiO_2_/Si; they were fabricated by using a Singulus ROTARIS Sputter, and cut into multiple 2 cm × 2 cm pieces. A 2 cm × 2 cm piece of the Ru/Ta/SiO_2_/Si substrate was cleaned by ultrasonication (model: KQ‐100KDB, power: 99 W), in acetone, alcohol, and deionized water for 15 min, respectively. The h‐BN (MoS_2_) flakes were then mechanically exfoliated from a bulk h‐BN (MoS_2_) crystal and transferred onto the clean Ru/Ta/SiO_2_/Si substrate by using a single side tape (SPV‐224PR, Nitto) with heat (60 °C for 3 min). The h‐BN crystals were from HQ graphene, and the MoS_2_ crystals were purchased from HQ Graphene (synthetic, with purity > 99.9995%).

### Fabrication of CVD‐Grown h‐BN

The CVD‐grown h‐BN samples, with thicknesses of 1.37 and 5.2 nm have been purchased from the company Alpha Chemistry. The 1.37‐nm‐thick sample belongs to the category “CVD‐grown single layer h‐BN” on the website of the manufacturer.^[^ ^49^ ^]^ However, it is not a monolayer, it is 1.37‐nm‐thick (or 4 layers), as confirmed by transmission electron microscopy (see Figure 7a). Inaccuracies of thickness and quality of CVD‐grown h‐BN from commercial sources are well known.^[^ ^26^ ^]^ The 5.2‐nm‐thick belongs to the category “CVD‐grown multilayer h‐BN” on the website of the manufacturer.^[^ ^50^ ^]^

### Characterization

Optical microscopy was performed using a Leica DM400 polarizing microscope. The CAFM characterization was carried out using a Dimension Icon AFM from Bruker, equipped with a Nanoscope VI controller and a PFTUNA module, under 22 °C. Two modes are used during the measurement: i) tapping mode under ambient atmosphere (RH = 54%), with an NCHV‐A Si tip (*k* = 40 N m^−1^); and ii) contact mode under different humidity environment (54, 46, 40, 30, 20, 10, and 4%), with two types of probes: i) RMN25PT300B, solid Pt tip (*R_tip_* = 25 nm, *k* = 18 N m^−1^), (b) CONTV‐PT, Pt‐coated Si tip (*R_tip_* = 25 nm, *k* = 0.2 N m^−1^). The solid Pt tip was used for the SiO_2_ samples and the CVD‐grown h‐BN samples, and the Pt‐coated Si tips were used for the mechanically exfoliated samples. The scan rate during lateral scans was 1 Hz. All the *I–* *V* curves are collected with a current limitation of 110 pA. The high‐resolution cross‐sectional TEM images were collected by using an FEI Titan ST S/TEM, operated under 200 kV. The FIB used to prepare the sample for the TEM experiment is a Helios G4 UX.

To modulate humidity, we introduce a tube in the CAFM chamber (see Figure S22a,b, Supporting Information) and flow dry nitrogen (N_2_, purity 99.9999%) while monitoring humidity levels with two sensors (see Figure S22c, Supporting Information). Upon reaching the target humidity, the N_2_ valve is promptly closed, followed by a 10‐min stabilization period before commencing measurements. During measurements at humidity levels above RH = 20% (e.g., 54%, 46%, 40%, and RH = 30%), the N_2_ valve remained fully closed to minimize the impact of N_2_ flow within the chamber (which may lead to tip vibration during the measurement). However, at lower RH levels (e.g., RH = 20%, RH = 10%, and RH = 4%), a slight N_2_ flow is necessary to maintain stable humidity levels throughout the experiment; failure to do so would result in a general increase in humidity levels during measurements, rendering the results unreliable. In this case, it was also to wait 10‐min for humidity stabilization before carrying out the measurements under RH = 20%, 10%, and 4%.

## Conflict of Interest

The authors declare no conflict of interest.

## Author Contributions

M.L. and Y.Y. conceived the idea and designed the experiments. Y.Y. performed all the experiments, including sample preparation and statistical characterization. M.L. and Y.Y. wrote the manuscript.

## Supporting information

Supporting Information

## Data Availability

The data that support the findings of this study are available from the corresponding author upon reasonable request.
