# Supplementary material for: The Effect of Relative Humidity in Conductive Atomic Force Microscopy
Source: Adv Mater. 2024 Sep 11;36(51):2405932. doi: 10.1002/adma.202405932 (PMC11656041; doi:10.1002/adma.202405932)
Supplement: Supplementary file 1 — Supporting Information [file ADMA-36-2405932-s001.docx]

**SUPPLEMENTARY INFORMATION**

**Effect of humidity in conductive atomic force microscopy**

Yue Yuan, Mario Lanza^*^

Materials Science and Engineering Program, Physical Science and Engineering Division,

King Abdullah University of Science and Technology (KAUST), Thuwal 23955, Saudi Arabia

^*^ Corresponding author Email: mario.lanza@kaust.edu.sa

**List of Figures**

Figure S1. Variability of solid Pt CAFM tips (RMN25PT300B).

Table S1. History of experiments carried out with each CAFM tip.

Figure S2. High-resolution cross-sectional TEM characterization on SiO_2_ sample.

Figure S3. CAFM topography maps on SiO_2_ / n^++^Si sample before and after *I*-*V*s.

Figure S4. CAFM RVS characterization on 3.4-nm-thick SiO_2_ / n^++^Si sample.

Figure S5. Schematic of the CAFM setup and *I*-*V* plots.

Figure S6. Estimating the correct *DS* in a CAFM study under *RH*=54%.

Figure S7. Estimating the correct *DS* in a CAFM study under *RH*=4%.

Figure S8. CAFM on SiO_2_/n^++^Si samples – confirmation of tip condition – *DS*=0.8V.

Figure S9. CAFM RVS characterization on 1.5-nm-thick SiO_2_ / n^++^Si sample.

Figure S10. CAFM RVS characterization on 2.3-nm-thick SiO_2_ / n^++^Si sample.

Figure S11. CAFM RVS characterization on 5.6-nm-thick SiO_2_ / n^++^Si sample.

Figure S12. CAFM on SiO_2_/n^++^Si samples – confirmation of tip condition.

Figure S13. CAFM current maps on 1.5-nm-thick SiO_2_ / n^++^Si.

Figure S14. CAFM topography maps on 1.5-nm-thick SiO_2_ / n^++^Si.

Figure S15. Determination of ratio between currents registered at *RH*=54 and 4%.

Note S1. Calculation of tip/sample contact area.

Figure S16. High-resolution cross-sectional TEM characterization on h-BN samples.

Figure S17. CAFM on mechanically exfoliated h-BN – confirmation of tip condition.

Figure S18. CAFM on CVD-grown multilayer h-BN – confirmation of tip condition.

Figure S19. CAFM on mechanically exfoliated MoS_2_ – confirmation of tip condition.

Figure S20. Current instabilities occasionally detected at *RH*=4%.

Figure S21. Dependence between scan frequency and *A_eff_*.

Figure S22. Photos of CAFM chamber with environment (humidity) control system.

**List of Figures**

**
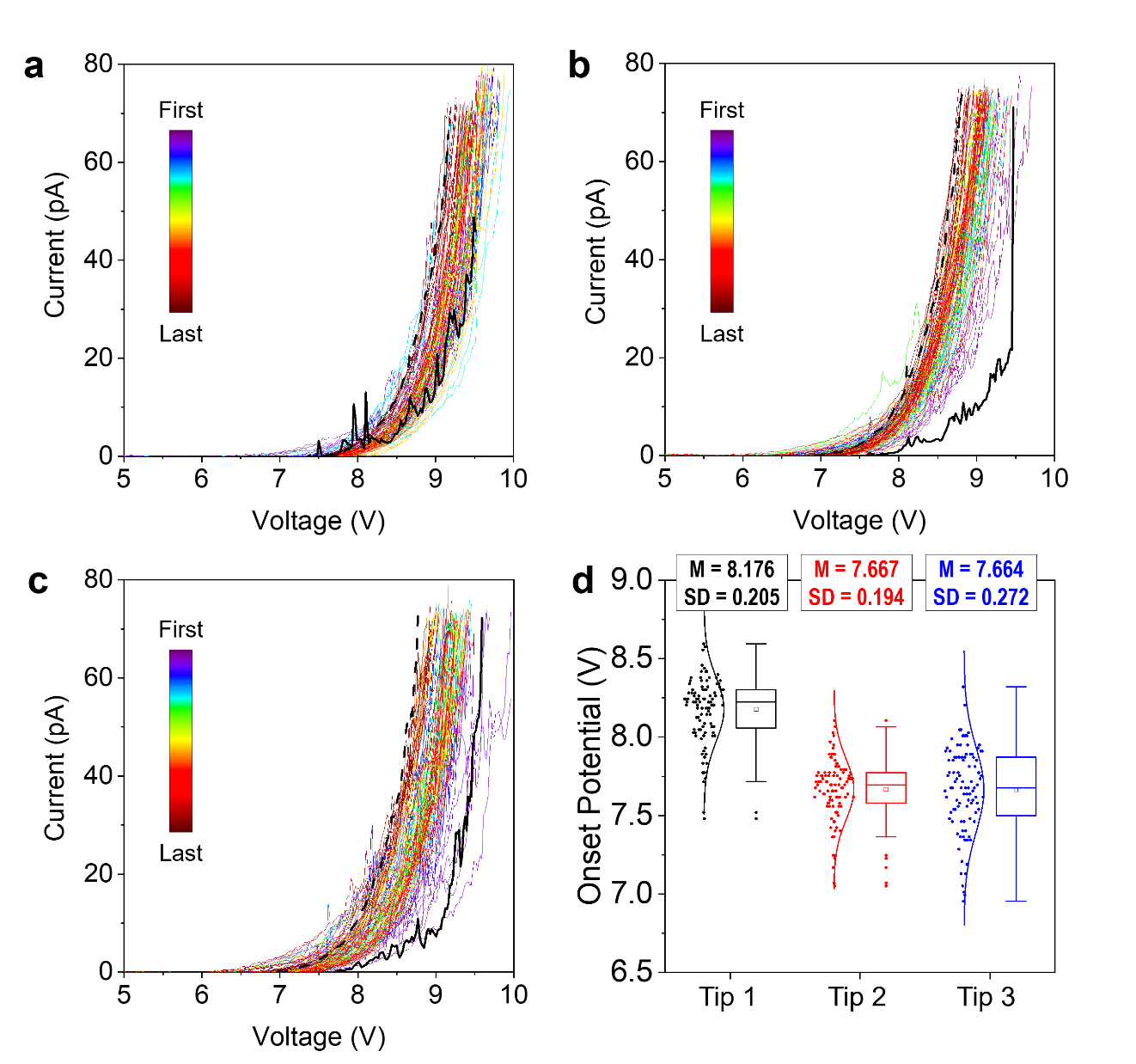
**

**Figure S1 | Variability of solid Pt CAFM tips (RMN25PT300B)**. **a**-**c**, Forward *I*-*V* curves recorded at 100 different locations of a 5.6 nm SiO_2_/n^++^Si sample using three different solid Pt tips (RMN25PT300B). The thicker black solid lines correspond to the first *I*-*V* curves, the black dashed lines correspond to the last *I*-*V* curves; this is to show that no tip degradation (i.e., downward trend) is observed. **d**, Statistical analysis of *V_ON_* (extracted at 3 pA). *M* and *SD* represent the mean value and the standard deviation of the distribution. Reproduced with permission from J. Weber et al. ACS Applied Materials and Interfaces, 15, 21602-21608 (2023). Copyright American Chemical Society (2023). These experiments clearly display that in CAFM science one should always try to measure all the samples with the same CAFM tip, trying to avoid tip wearing (during experiments) as much as possible. This was extremely complex in the past due to the fast wearing of the metal-coated Si tips CAFM tips, but the recent development of solid Pt tips combined with the current limitation system integrated in the Bruker Dimension Icon CAFM makes for the first time such extremely accurate characterization possible.

| **Tip 1**  **RMN25PT300B** | **Number of**  ***I*-*V* curves** | **Purpose / Observation** | **Figure Number** |
| --- | --- | --- | --- |
| 3.4 nm SiO_2_/n++Si | 1200 | Test: *RH*=54%, 12 *DS*, 100 *I*-*V*s at each | Figure 1 |
| 3.4 nm SiO_2_/n++Si | 1200 | Test: *RH*=4%, 12 *DS*, 100 *I*-*V*s at each | Figure 3 |
| **Total** | **2400** | **No tip degradation** |  |
|  |  |  |  |
| **Tip 2**  **RMN25PT300B** | **Number of**  ***I*-*V* curves** | **Purpose / Observation** | **Figure Number** |
| 3.4 nm SiO_2_/n++Si | 2,100 | Test: All 7 *RH*, 3 areas at each, 100 *I*-*V*s at each | Figures 2, 4 |
| 3.4 nm SiO_2_/n++Si | 100 | Integrity: *RH*=54%, 1 area, 100 *I*-*V*s | Figure S12 |
| 5.6 nm SiO_2_/n++Si | 2,100 | Test: All 7 *RH*, 3 areas at each, 100 *I*-*V*s at each | Figure 4, Figure S11 |
| 5.6 nm SiO_2_/n++Si | 100 | Integrity: *RH*=54%, 1 area, 100 *I*-*V*s | Figure S12 |
| 1.37 nm CVD h-BN | 700 | Test: All 7 *RH*, 1 area at each, 100 *I*-*V*s at each | Figure 7 |
| 1.37 nm CVD h-BN | 100 | Integrity: *RH*=54%, 1 area, 100 *I*-*V*s | Figure 7 |
| 5.2 nm CVD h-BN | 700 | Test: All 7 *RH*, 1 area at each, 100 *I*-*V*s at each | Figure 7 |
| 5.2 nm CVD h-BN | 100 | Integrity: *RH*=54%, 1 area, 100 *I*-*V*s | Figure 7 |
| **Total** | **6,000** | **No tip degradation** |  |
|  |  |  |  |
| **Tip 3**  **RMN25PT300B** | **Number of**  ***I*-*V* curves** | **Purpose / Observation** | **Figure Number** |
| 1.5 nm SiO_2_/n++Si | 2,100 | Test: All 7 *RH*, 3 areas at each, 100 *I*-*V*s at each | Figure 4, Figure S9 |
| 1.5 nm SiO_2_/n++Si | 100 | Integrity: *RH*=54%, 1 area, 100 *I*-*V*s | Figure S12 |
| **Total** | **2,200** | **No tip degradation** |  |
|  |  |  |  |
| **Tip 4**  **RMN25PT300B** | **Number of**  ***I*-*V* curves** | **Purpose / Observation** | **Figure Number** |
| 2.3 nm SiO_2_/n++Si | 2,100 | Test: All 7 *RH*, 3 areas at each, 100 *I*-*V*s at each | Figure 4, Figure S10 |
| 2.3 nm SiO_2_/n++Si | 100 | Integrity: *RH*=54%, 1 area, 100 *I*-*V*s | Figure S12 |
| Ru | 200 | Test: *RH*=54% and 4%, 1 area, 100 *I*-*V*s at each | Figure 9 |
| Ru | 100 | Integrity: *RH*=54%, 1 area, 100 *I*-*V*s | Figure 9 |
| **Total** | **2,500** | **No tip degradation** |  |
|  |  |  |  |
|  |  | *Continue in next page* |  |
| **Tip 5**  **CONTV-Pt** | **Number of**  ***I*-*V* curves** | **Purpose / Observation** | **Figure Number** |
| 3.32 nm ME h-BN | 700 | Test: All 7 *RH*, 1 area at each, 100 *I*-*V*s at each | Figure 6 |
| 3.32 nm ME h-BN | 100 | Integrity: *RH*=54%, 1 area, 100 *I*-*V*s | Figure 6 |
| 1.88 nm ME MoS_2_ | 700 | Test: All 7 *RH*, 1 area at each, 100 *I*-*V*s at each | Figure 8 |
| 1.88 nm ME MoS_2_ | 100 | Integrity: *RH*=54%, 1 area, 100 *I*-*V*s | Figure 8 |
| 8.66 nm ME MoS_2_ | 700 | Test: All 7 *RH*, 1 area at each, 100 *I*-*V*s at each | Figure 8 |
| 8.66 nm ME MoS_2_ | 100 | Integrity: *RH*=54%, 1 area, 100 *I*-*V*s | Figure 8 |
| **Total** | **2,400** | **No tip degradation** |  |
|  |  |  |  |
| **Tip 6**  **CONTV-Pt** | **Number of**  ***I*-*V* curves** | **Purpose / Observation** | **Figure Number** |
| 3.4 nm SiO_2_/n++Si | 1200 | Test: *RH*=54%, 12 *DS*, 100 *I*-*V*s at each | Figure S6 |
| 3.4 nm SiO_2_/n++Si | 25 | Integrity: *RH*=54%, 1 area, 25 *I*-*V*s | Figure S8 |
| 3.4 nm SiO_2_/n++Si | 300 | Test: *RH*=4%, 12 *DS*, 25 *I*-*V*s at each | Figure S7 |
| 3.4 nm SiO_2_/n++Si | 25 | Integrity: *RH*=4%, 1 area, 25 *I*-*V*s | Figure S8 |
| **Total** | **1550** | **No tip degradation** |  |

**Table S1 | History of experiments carried out with each CAFM tip.** Detailed description of the experiments carried out with each CAFM tip used in this study to investigate the influence of the relative humidity in all the samples (insulating, semiconducting and conductive) via ramped voltage stresses (RVS). All CAFM tips keep perfect integrity without degradation, mainly thanks to the 110 pA current limitation used during the RVS. In total, 17,050 *I*-*V* curves have been carried out. Other RVS aimed to determine the suitable deflection setpoint and to evaluate tip-to-tip variability are not included. Current maps are also not included here.


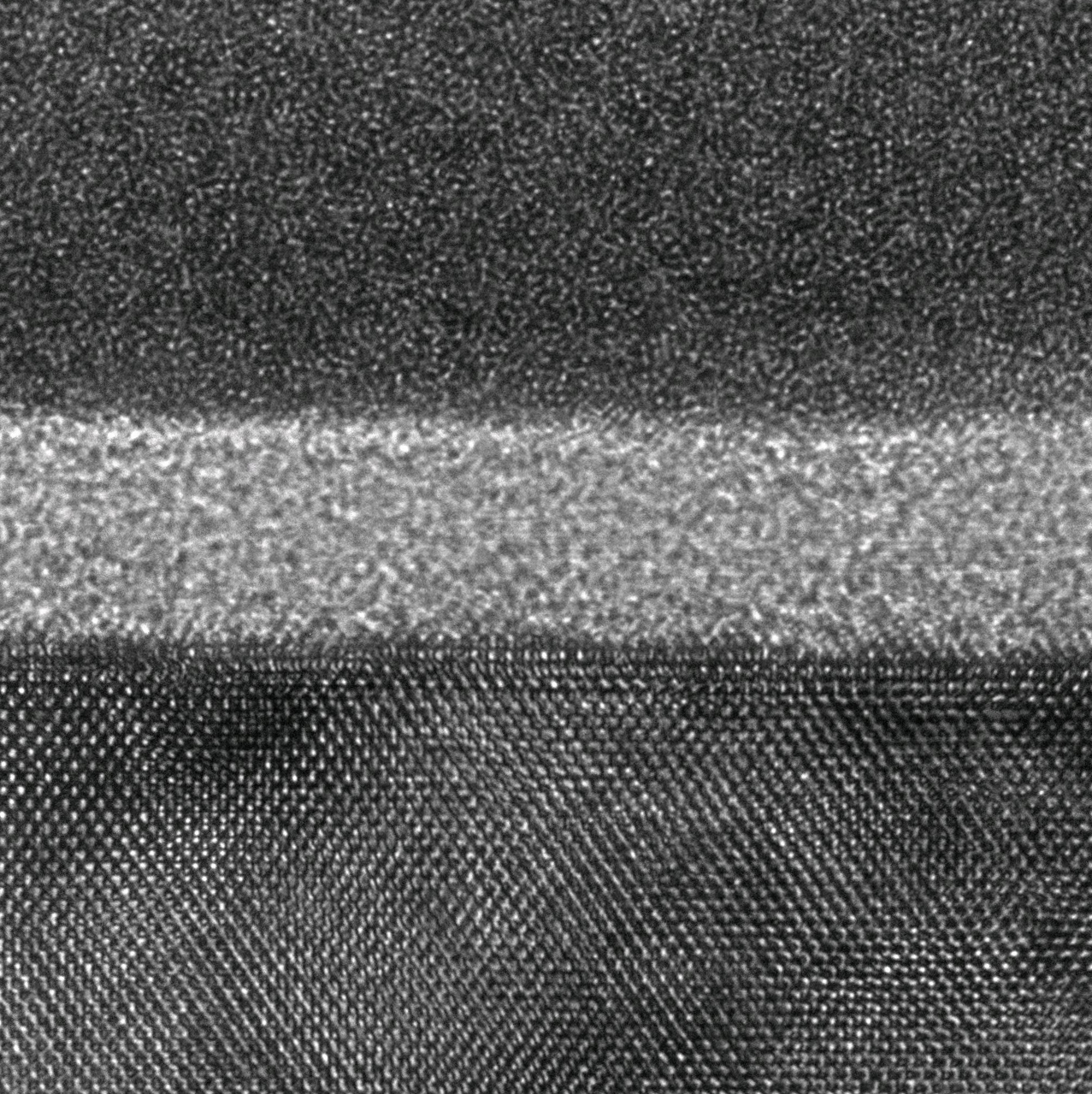


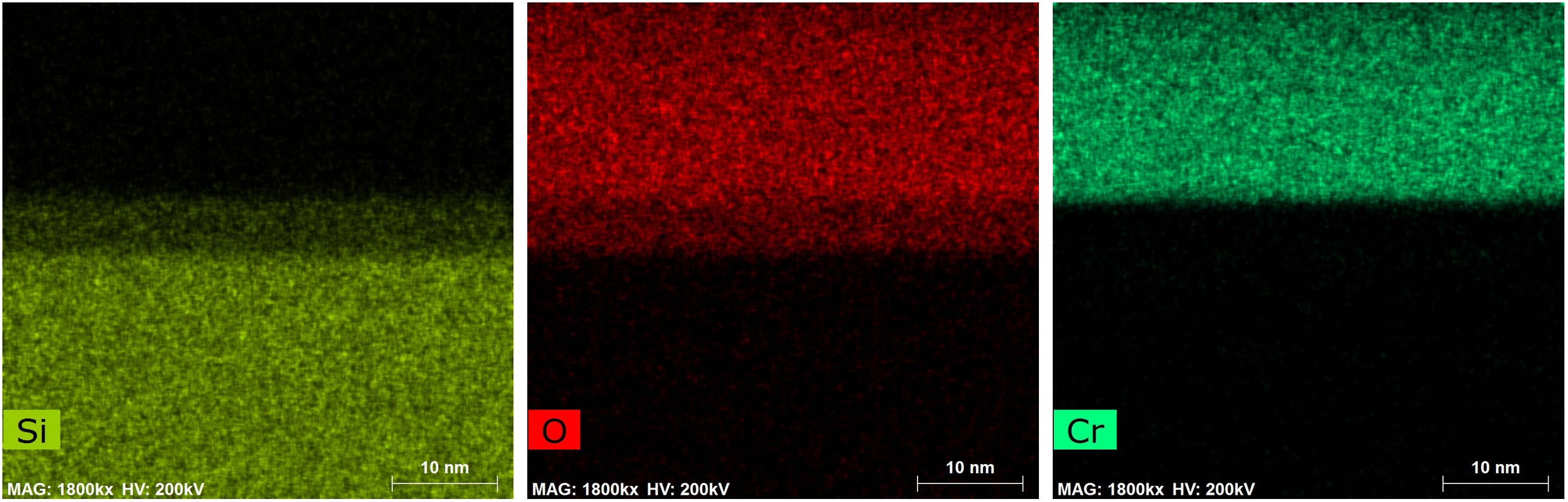


**Figure S2 | Cross-sectional TEM of a SiO_2_/n^++^Si sample.** The image shows the crystalline lattice of the Si film and the amorphous SiO_2_ film. The fabrication method was thermal oxidation, which results in SiO_2_ films very homogeneous and with very low thickness fluctuations. The bottom row shows the energy dispersive X-ray spectroscopy maps of the sample, displaying its chemical composition. The top Cr layer is used for protection in the cross-sectional TEM analysis, and it is not present in the CAFM study.


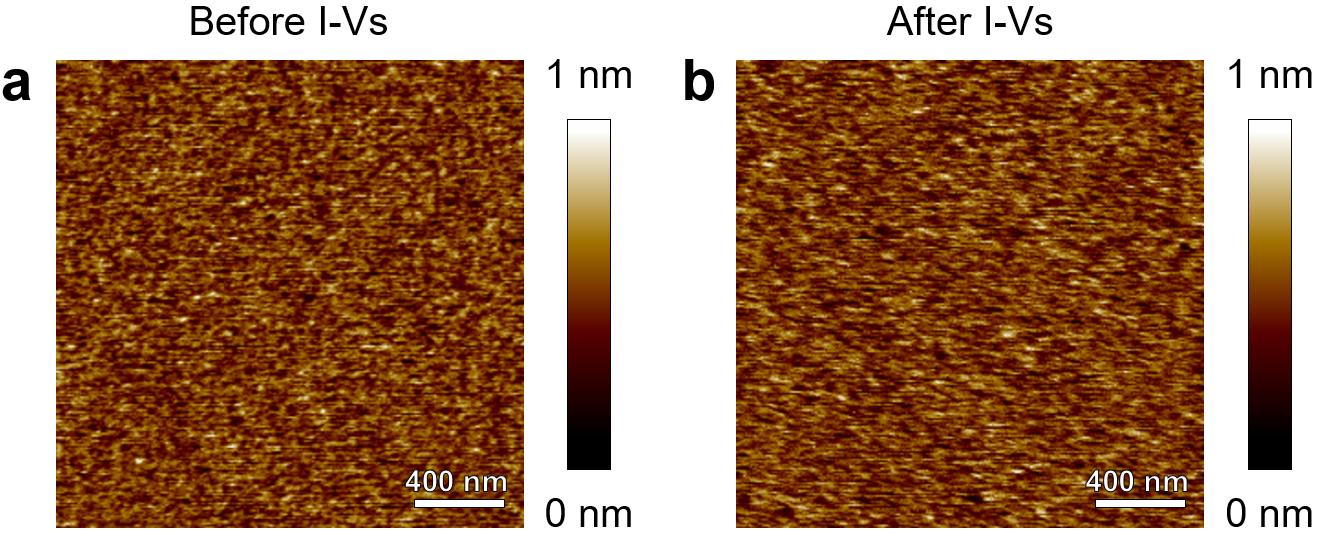


**Figure S3 | CAFM topography maps on 3.4-nm-thick SiO_2_ / n^++^Si sample before and after *I*-*V* measurements. a**, CAFM topography map before *I*-*V*. **b**, CAFM topography map after *I*-*V*s (same location as **a**).


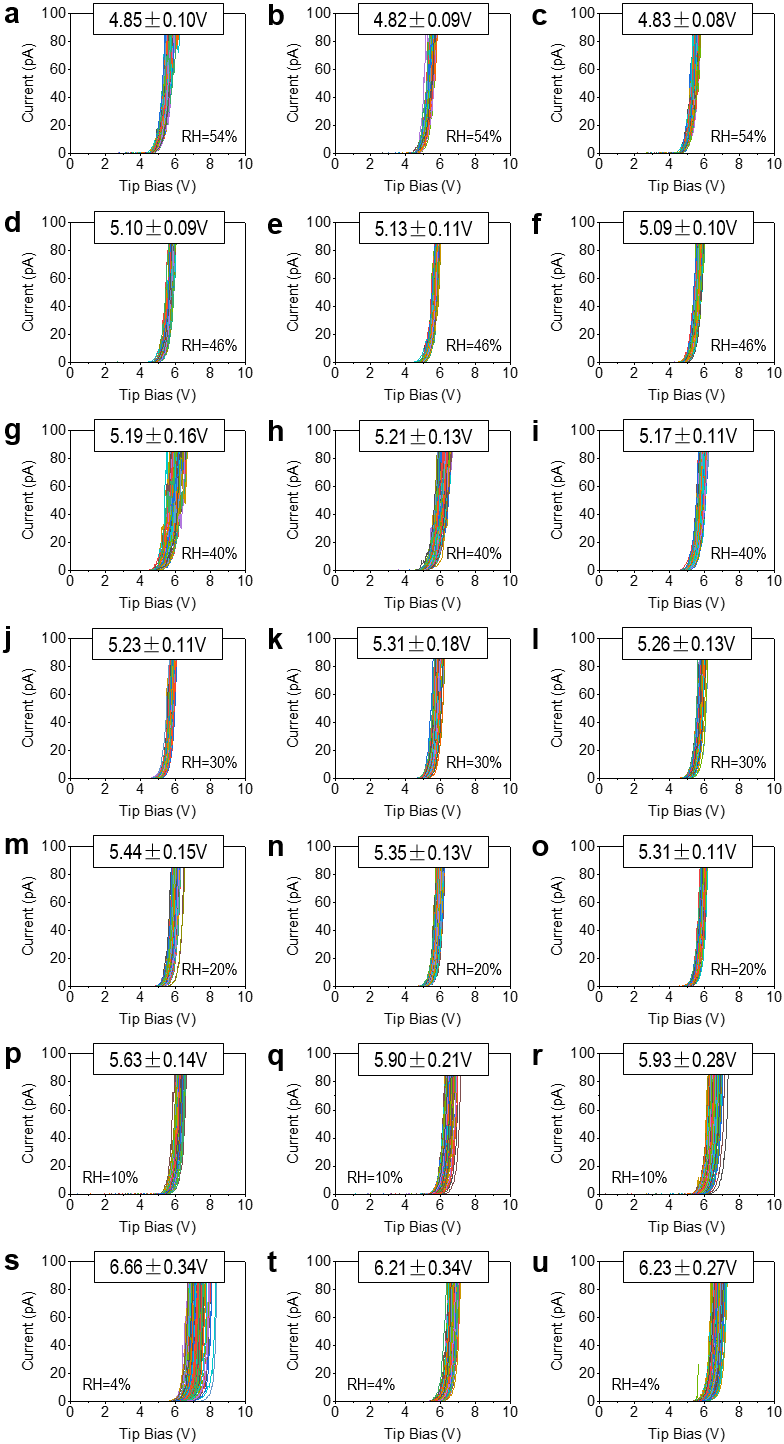


**Figure S4 | CAFM RVS characterization on 3.4-nm-thick SiO_2_ / n^++^Si sample.** Each *I*-*V* plot contains 100 *I*-*V* curves with a current limitation of 110 pA, and each collected at a different location. **a-c**, *I*-*V* curves collected under *RH*=54%, at three different locations. **d-f**, *I*-*V* curves collected under *RH*=46%. **g-i**, *I*-*V* curves collected under *RH*=40%. **j-l**, *I*-*V* curves collected under *RH*=30%. **m-o**, *I*-*V* curves collected under *RH*=20%. **p-r**, *I*-*V* curves collected under *RH*=10%. **s-u**, *I*-*V* curves collected under *RH*=4%.


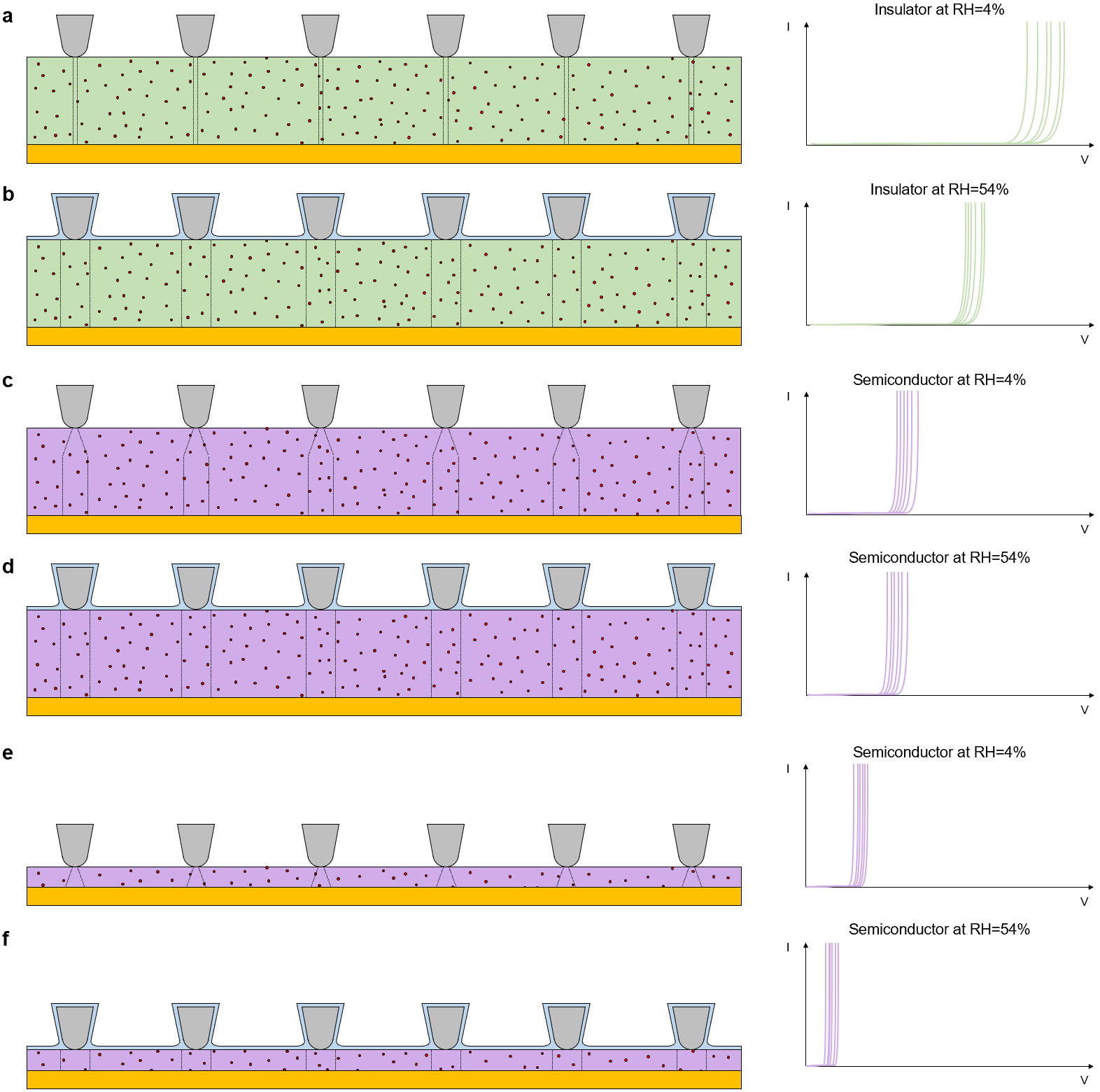


**Figure S5 | Schematic of the CAFM setup and *I*-*V* plots in each experimental condition for different samples.** The grey semicircles represent the CAFM tip at different positions of the sample. The green material is insulating, the purple is semiconducting, and the grey and orange are conducting. The red balls represent atomic defects in the samples. The two dashed lines indicate the limits of *A_eff_*. The blue shape surrounding the grey CAFM tips indicate the presence of a water meniscus under *RH*=54%, while the schematics without it represent the setup under *RH*=4%. For a sample with a given density of defects (red balls), the number of defects inside *A_eff_* (between the two dashed lines) at six different positions presents a much higher variation. In particular, for a large *A_eff_* (*RH*=54%) the number of defects in panel **a** are 8, 7, 9, 9, 7, and 9 (i.e., 8.16 ± 0.98, *C_v_* = 12.0%), and for a small *A_eff_* (*RH*=4%) these numbers change into 1, 0, 4, 1, 0, and 3 (i.e., 1.50 ± 1.61, *C_v_* = 107.3%).


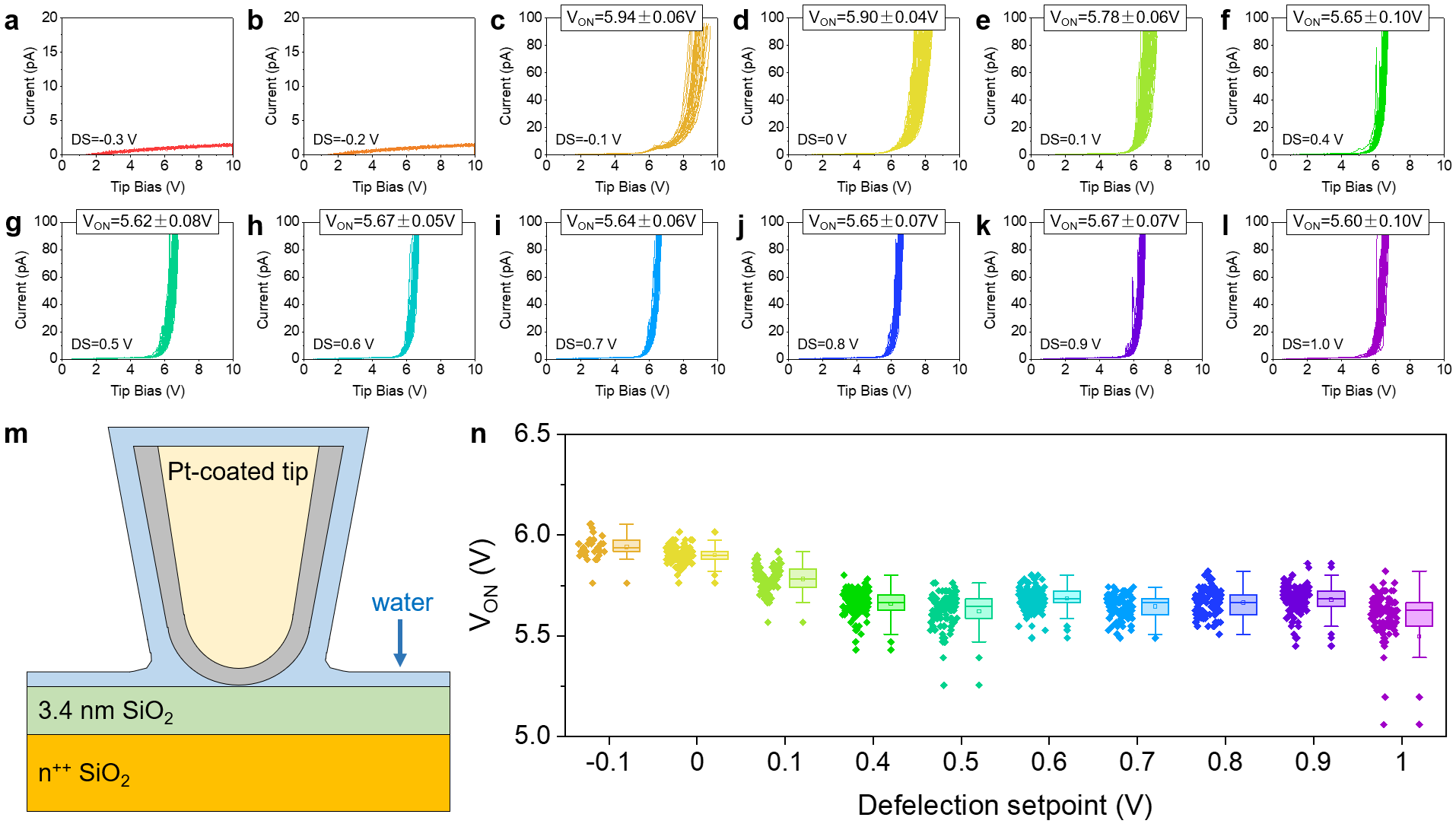


**Figure S6 | Estimating the correct *DS* in a CAFM study under *RH*=54%. a-l,** *I*-*V* curves collected with a CONTVPT Pt-coated Si tip (using a current limitation of 110 pA) on the surface of a 3.4 nm SiO_2_/n^++^Si sample, under different deflection setpoint voltages of -0.3, -0.2, -0.1, 0, 0.1, 0.4, 0.5, 0.6, 0.7, 0.8, 0.9, and 1.0 V, respectively (*RH*=54%). Each plot contains 100 *I*-*V* curves collected in a 10 × 10 matrix with point-to-point distances of 1 µm. **m,** Schematic of the CAFM setup under *RH*=54% when the Pt-coated Si tip is in contact with the surface of the sample. The grey semicircles represent the Pt-coated Si tip. The green and orange rectangles represent the sample, and the blue shape surrounding both tip and sample represents the water film at *RH*=54%. **n**, Statistical analysis on *V_ON_* versus deflection setpoint extracted from **c**-**l**.


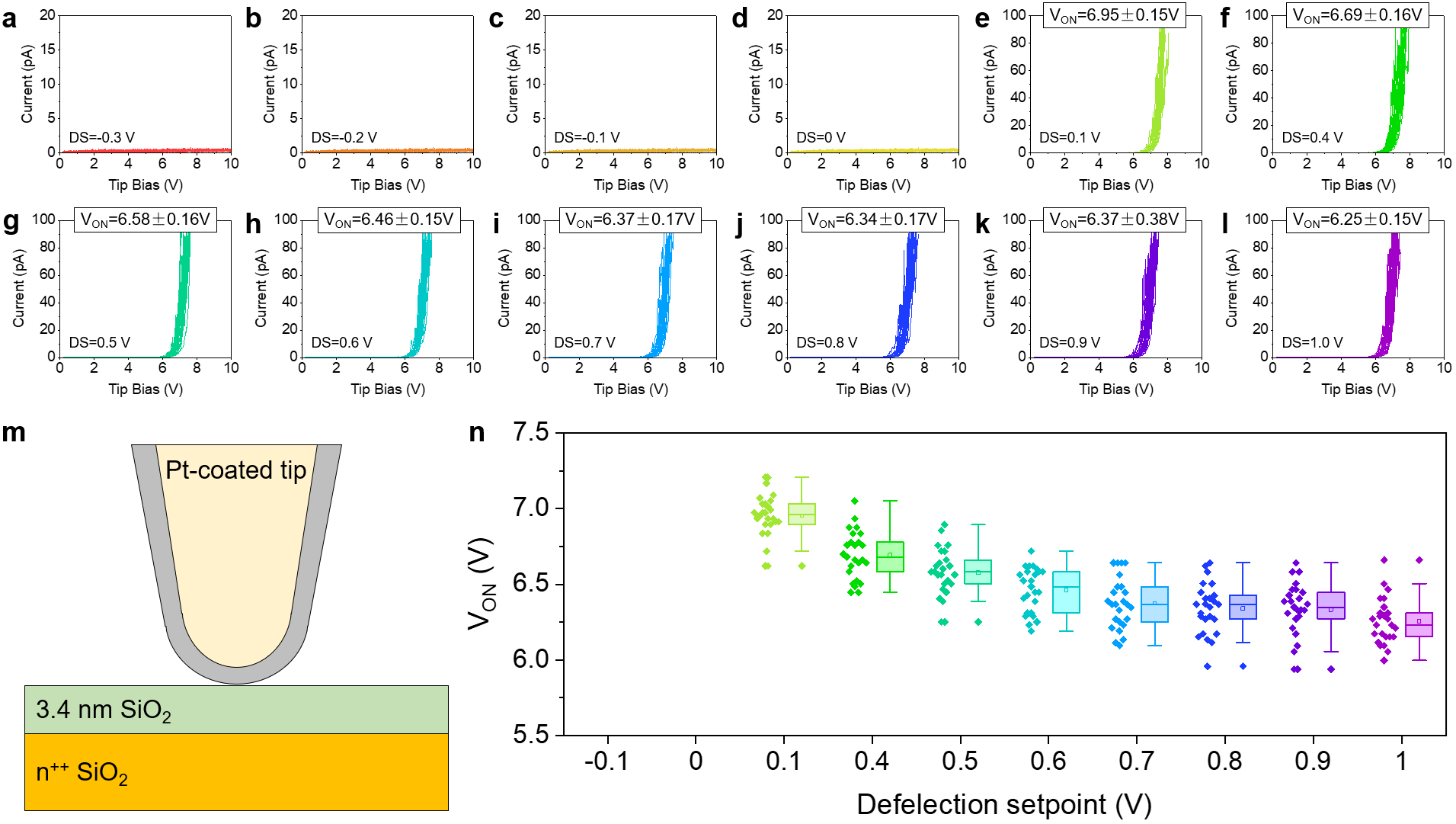


**Figure S7 | Estimating the correct *DS* in a CAFM study under *RH*=4%. a-l,** *I*-*V* curves collected with a CONTVPT Pt-coated Si tip (using a current limitation of 110 pA) on the surface of a 3.4 nm SiO_2_/n^++^Si sample, under different deflection setpoint voltages of -0.3, -0.2, -0.1, 0, 0.1, 0.4, 0.5, 0.6, 0.7, 0.8, 0.9, and 1.0 V, respectively (*RH*=4%). Each plot contains 100 *I*-*V* curves collected in a 10 × 10 matrix with point-to-point distances of 1 µm. **m,** Schematic of the CAFM setup under *RH*=4% when the Pt-coated Si tip is in contact with the surface of the sample. The grey semicircles represent the Pt-coated Si tip. The green and orange rectangles represent the sample. **n**, Statistical analysis on *V_ON_* versus deflection setpoint extracted from **c**-**l**.


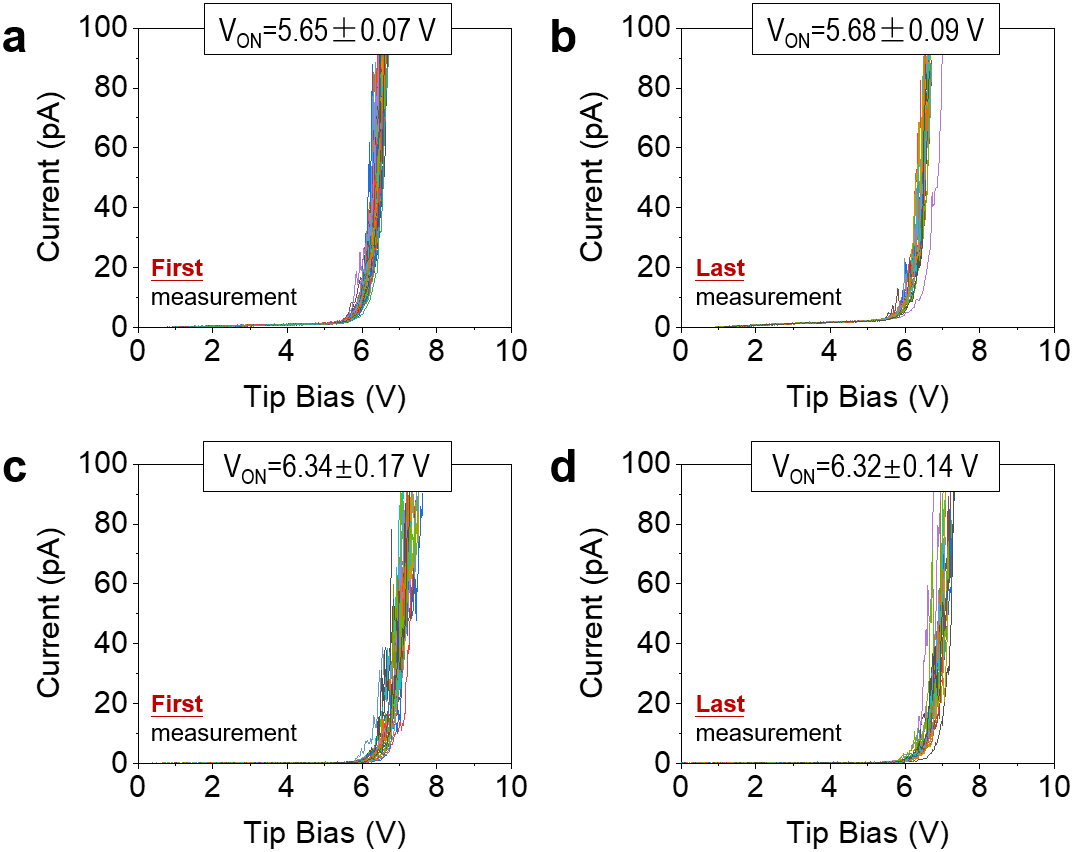


**Figure S8 | CAFM RVS characterization on 3.4 nm SiO_2_ / n^++^ Si samples under *RH*=54% and 4% – confirmation of tip condition. a**-**d**, all the *I*-*V* curves are measured with a CONTV-PT tip. **a** and **c** contain 100 *I*-*V* curves for each, with a current limitation of 110 pA, and each collected at a different location. **b** and **d** contain 25 *I*-*V* curves for each, with a current limitation of 110 pA, and each collected at a different location. **a-b**, The first and the last groups of *I*-*V* curves collected on 3.4-nm-thick SiO_2_ sample, respectively, by using a *DS*=0.8 V, under *RH*=54%. **b** is collected after Figure S6**l**. **c-d**, The first and the last groups of *I*-*V* curves collected on 3.4-nm-thick SiO_2_ sample, respectively, by using a *DS*=0.8 V, under *RH*=4% **d** is collected after Figure S7**l**.


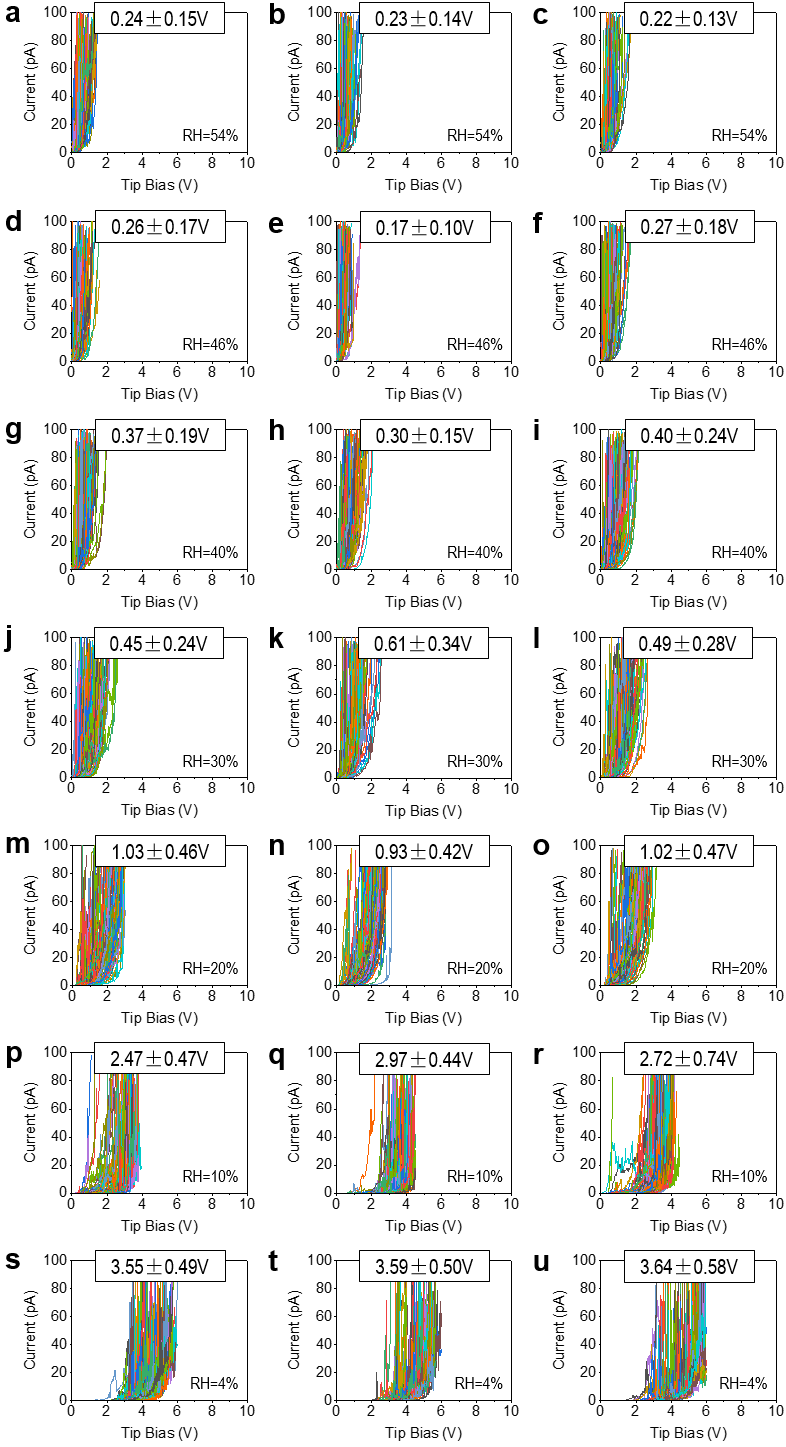


**Figure S9 | CAFM RVS characterization on 1.5-nm-thick SiO_2_ / n^++^Si sample.** Each *I*-*V* plot contains 100 *I*-*V* curves with a current limitation of 110 pA, and each collected at a different location. **a-c**, *I*-*V* curves collected under *RH*=54%, at three different locations. **d-f**, *I*-*V* curves collected under *RH*=46%. **g-i**, *I*-*V* curves collected under *RH*=40%. **j-l**, *I*-*V* curves collected under *RH*=30%. **m-o**, *I*-*V* curves collected under *RH*=20%. **p-r**, *I*-*V* curves collected under *RH*=10%. **s-u**, *I*-*V* curves collected under *RH*=4%.


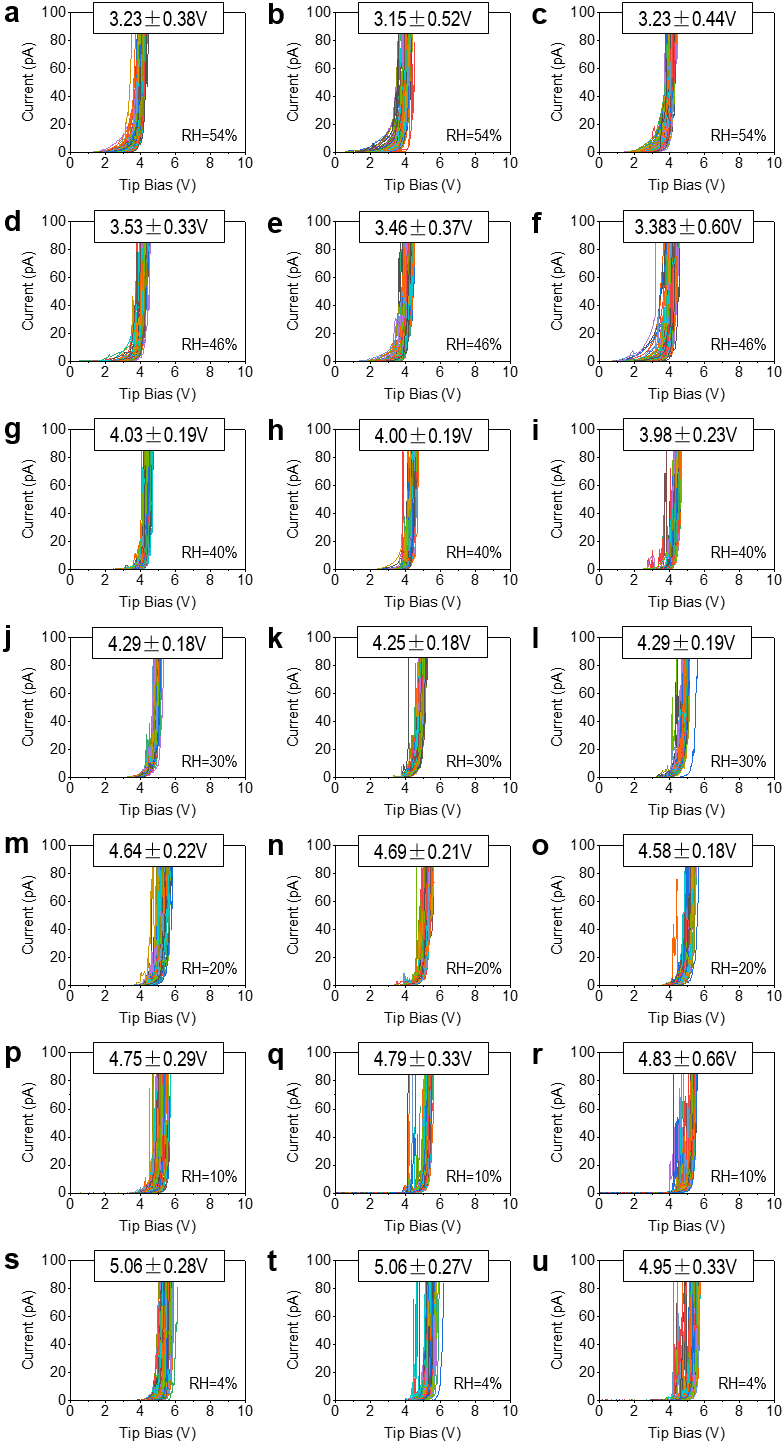


**Figure S10 | CAFM RVS characterization on 2.3-nm-thick SiO_2_ / n^++^Si sample.** Each *I*-*V* plot contains 100 *I*-*V* curves with a current limitation of 110 pA, and each collected at a different location. **a-c**, *I*-*V* curves collected under *RH*=54%, at three different locations. **d-f**, *I*-*V* curves collected under *RH*=46%. **g-i**, *I*-*V* curves collected under *RH*=40%. **j-l**, *I*-*V* curves collected under *RH*=30%. **m-o**, *I*-*V* curves collected under *RH*=20%. **p-r**, *I*-*V* curves collected under *RH*=10%. **s-u**, *I*-*V* curves collected under *RH*=4%.


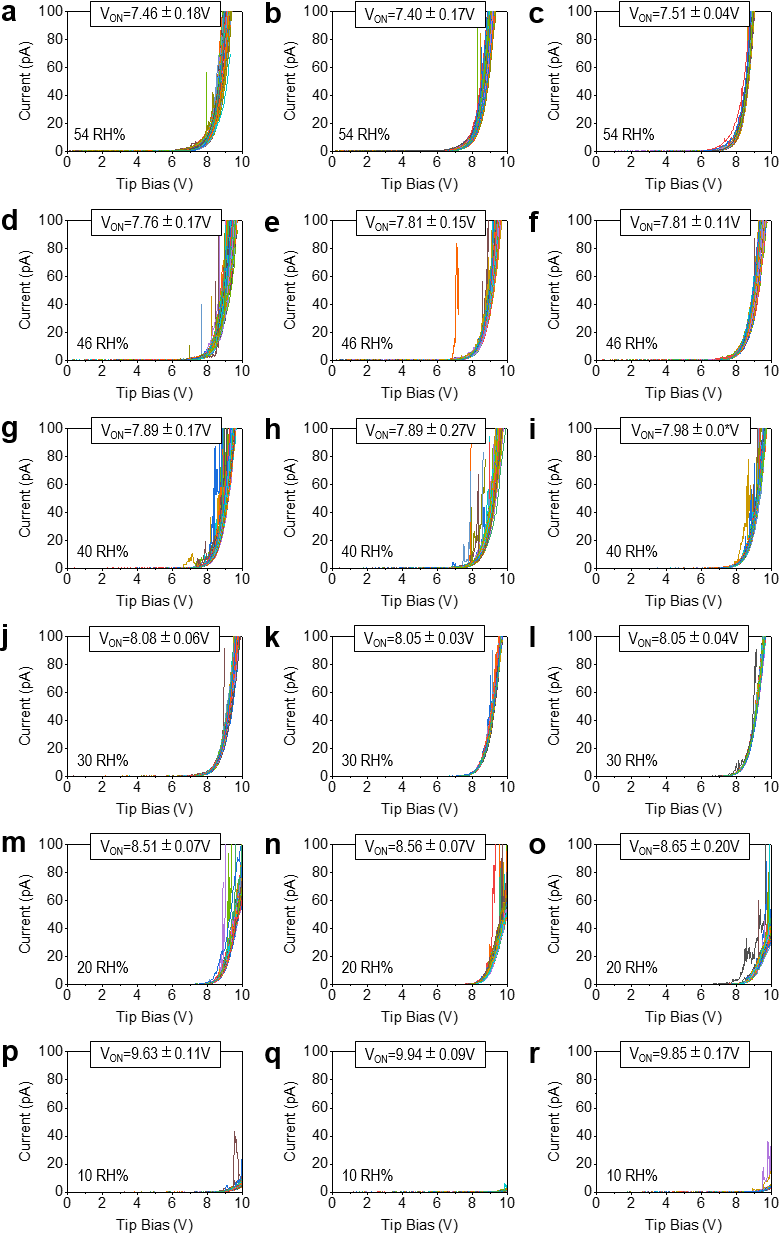


**Figure S11 | CAFM RVS characterization on 5.6-nm-thick SiO_2_ / n^++^Si sample.** Each *I*-*V* plot contains 100 *I*-*V* curves with a current limitation of 110 pA, and each collected at a different location. **a-c**, *I*-*V* curves collected under *RH*=54%, at three different locations. **d-f**, *I*-*V* curves collected under *RH*=46%. **g-i**, *I*-*V* curves collected under *RH*=40%. **j-l**, *I*-*V* curves collected under *RH*=30%. **m-o**, *I*-*V* curves collected under *RH*=20%. **p-r**, *I*-*V* curves collected under *RH*=10%.


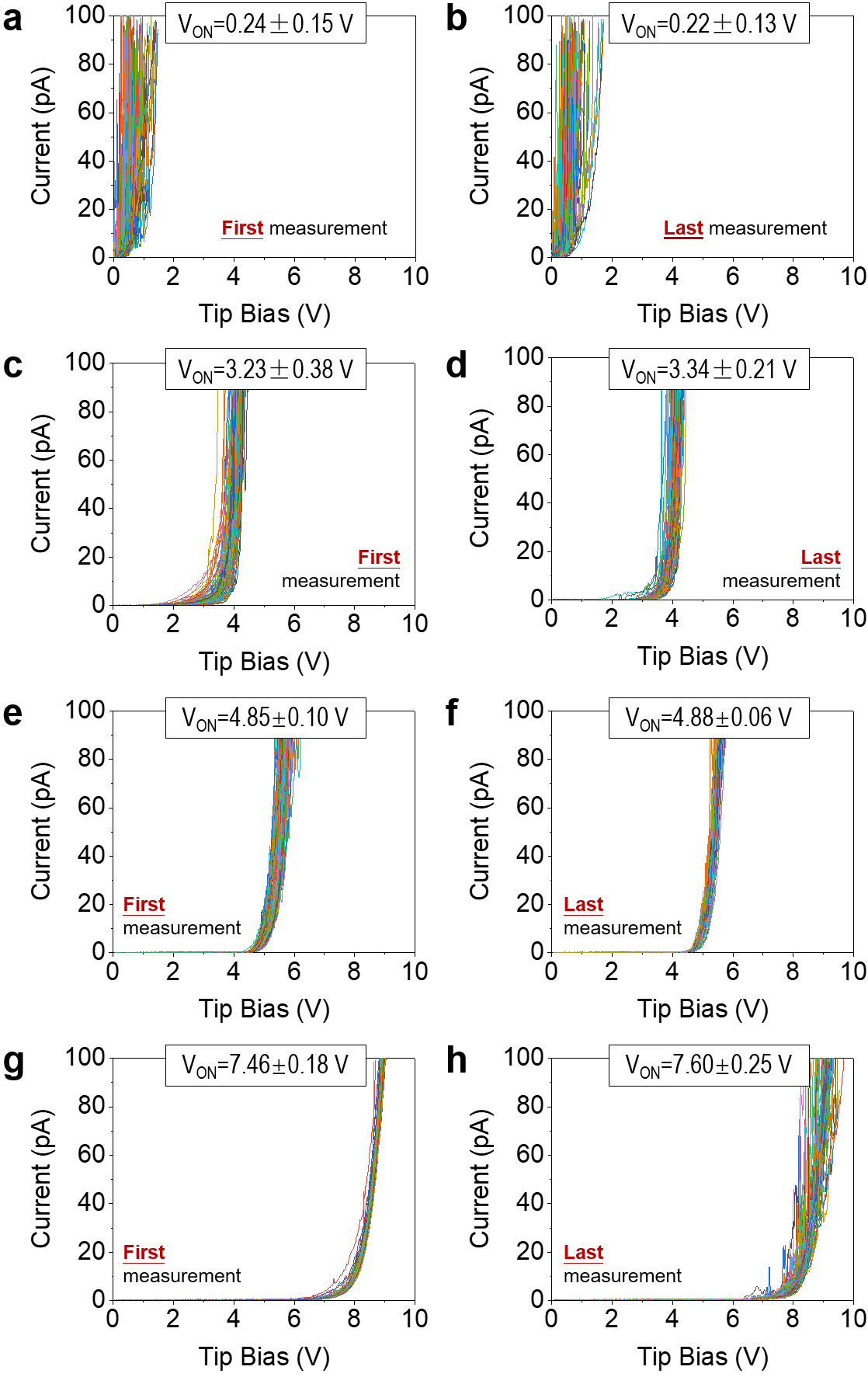


**Figure S12 | CAFM RVS characterization on SiO_2_ / n^++^ Si samples in air (*RH*=54%) – confirmation of tip condition.** Each *I*-*V* plot contains 100 *I*-*V* curves with a current limitation of 110 pA, and each collected at a different location. **a-b**, The first and the last groups of *I*-*V* curves collected on 1.5-nm-thick SiO_2_ sample, respectively. **b** is collected after Figure S9**u**. **c-d**, The first and the last groups of *I*-*V* curves collected on 2.3-nm-thick SiO_2_ sample, respectively. **d** is collected after Figure S10**u**. **e-f**, The first and the last groups of *I*-*V* curves collected on 3.4-nm-thick SiO_2_ sample, respectively. **f** is collected after Figure S4**u**. **g-h**, The first and the last groups of *I*-*V* curves collected on 5.6-nm-thick SiO_2_ sample, respectively. **h** is collected after Figure S11**r**.


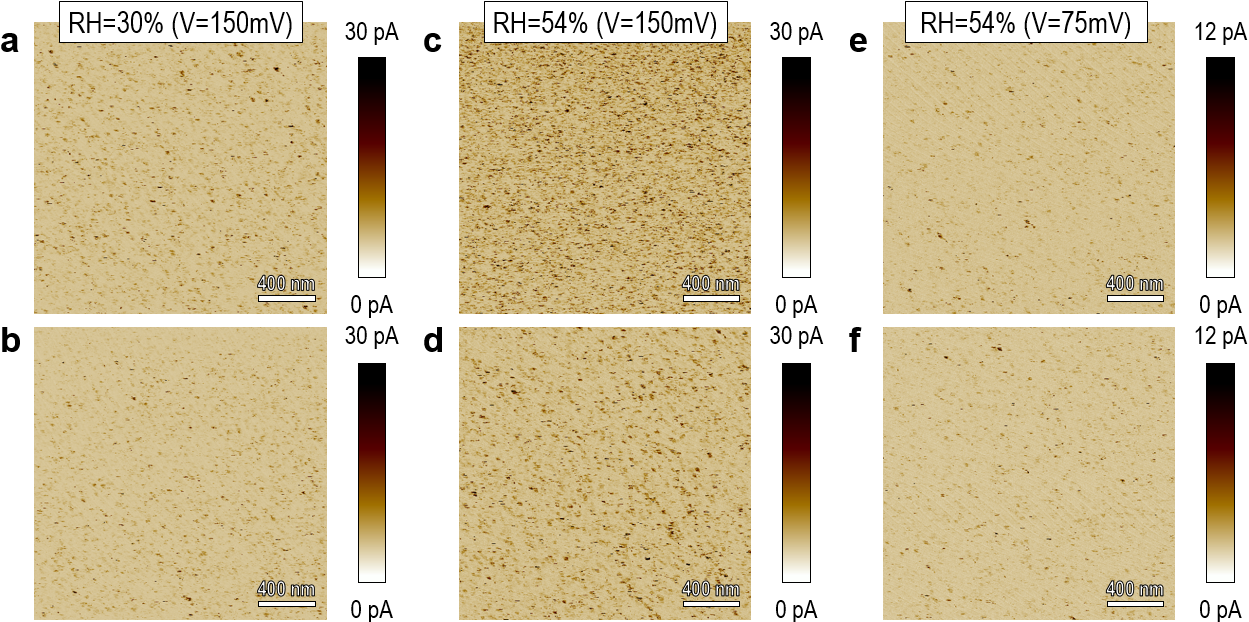


**Figure S13 | CAFM current maps on 1.5-nm-thick SiO_2_ / n^++^Si.**  These six CAFM current maps are collected at different locations. a-b, CAFM current maps collected under *RH*=30%, with a voltage of 150 mV (V1). c-d, CAFM current maps collected under *RH*=54% (in air), with a voltage of 150 mV (V1). e-f, CAFM current maps collected under *RH*=54% (in air), with a voltage of 75 mV (V2).


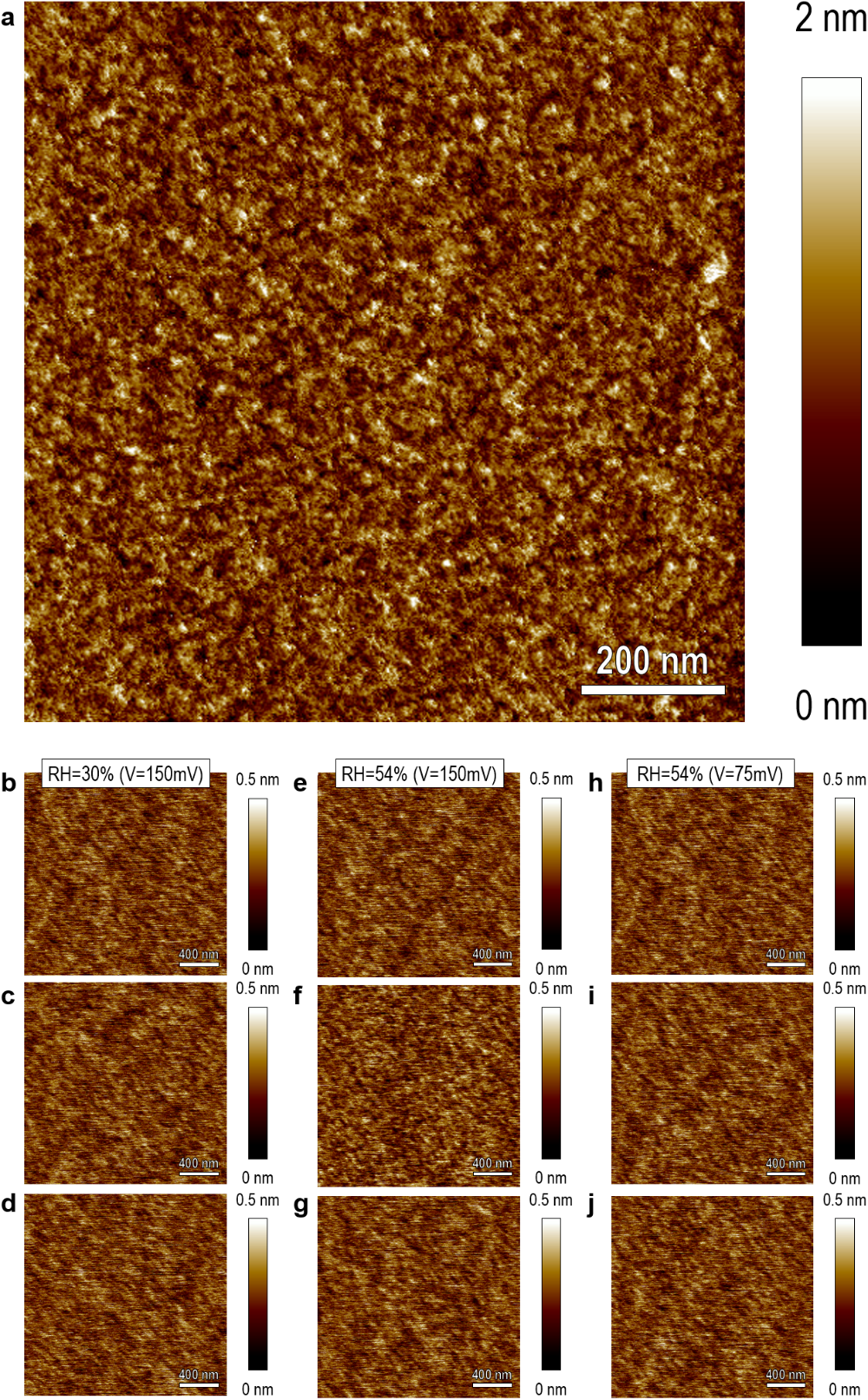


**Figure S14 | CAFM topography maps on 1.5-nm-thick SiO_2_ / n^++^Si.** **a,** AFM topography map collected by using Si tip under tapping mode. **b-j,** These nine CAFM topography maps are collected at different locations and simultaneously with the current maps presented in Figure 5b-d and Figure S13. **b-d,** CAFM topography maps collected under *RH*=30%, with a voltage of 150 mV (V1). **e-g,** CAFM topography maps collected under *RH*=54%, with a voltage of 150 mV (V1). **h-j,** CAFM topography maps collected under *RH*=54%, with a voltage of 75 mV (V2).


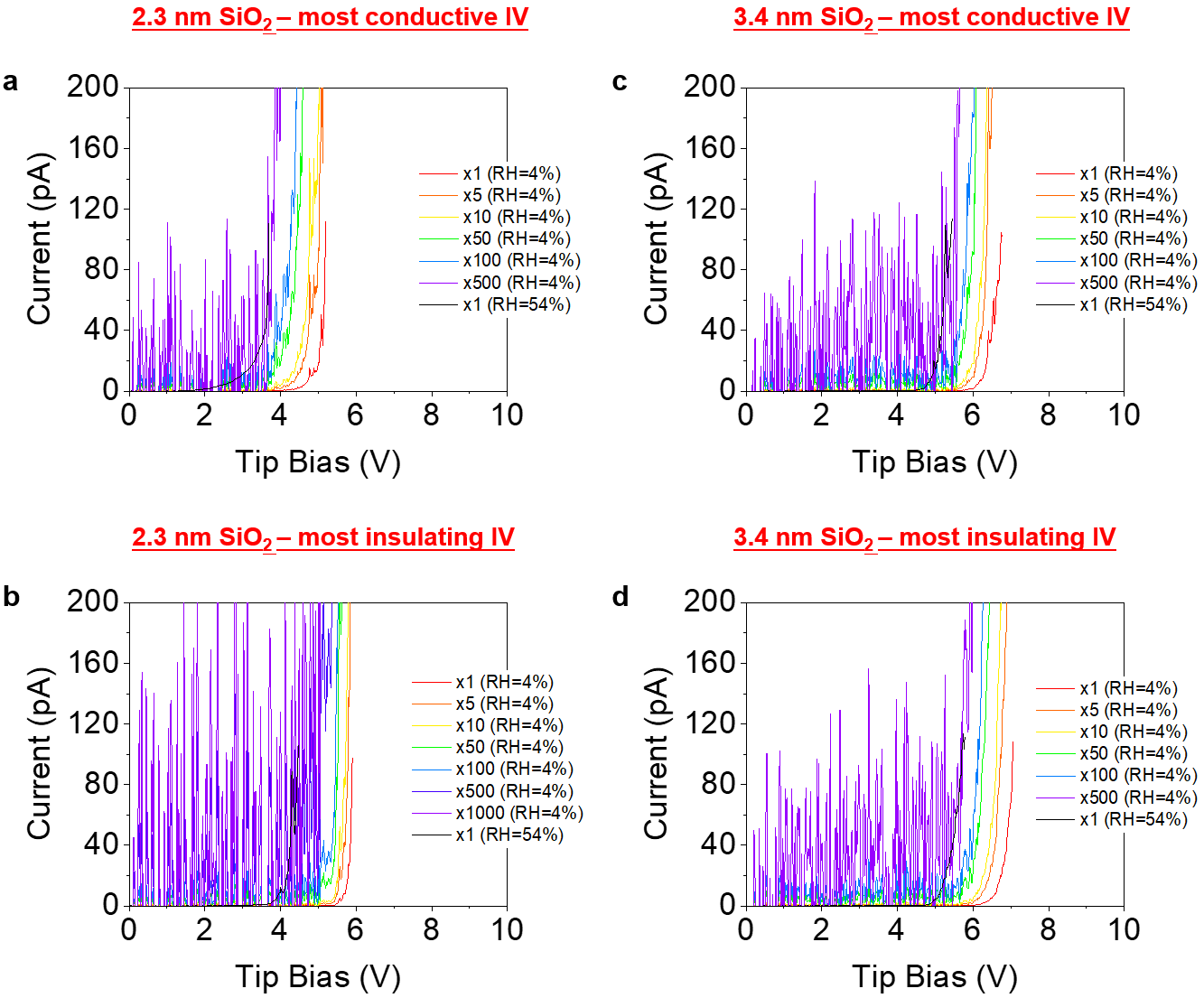


**Figure S15 | Determination of the ratio between the currents registered at *RH*=54% and *RH*=4%.** Typical *I*-*V* sweep measured for the 2.3 nm SiO_2_/n^++^Si sample at *RH*=4% and multiplied for a factor until matching the typical value measured for the same sample at *RH*=54%. When multiplying by high numbers the noise is also amplified, but comparisons of currents above 80 pA are still meaningful and can give an indication of the increase of current produced by the water meniscus.

**Note S1 | Calculation of tip/sample contact area (*A_c_*)**

In this note S1 we show how we have calculated the value of *A_c_*, although this has been also reported in other previous studies. When using more than one tip to characterize a group of samples

**Equations:**

$$r_{c}=\sqrt[3]{\frac{F_{c}\times R_{tip}}{K}}$$

$$\frac{1}{K}=\frac{3}{4}\left( \frac{1-\gamma_{1}^{2}}{E_{1}}+\frac{1-\gamma_{2}^{2}}{E_{2}} \right)$$

$$A_{c}=\pi\times r_{c}^{2}$$

Where,

*r_c_* is the contact radius;

*F_c_* is the contact force;

*E_1_* and *E_2_* are the elasticity modulus;

*γ_1_* and *γ_2_* are the Poisson ratios of the tip and the sample;

*A_c_* is contact area between the tip and the sample.

**Reference data**:

Solid Pt tip (Platinum):

Elasticity modulus $E_{1}=170 GPa=170\times{10}^{9} Pa$

Poisson ratio $\gamma_{1}=0.39$

[Data from: Elasticity modulus and Poisson ratio of Pt reproduced with permission from Brandes, E. A., & Brook, G. B. (Eds.). Smithells metals reference book. Elsevier (2013).]

Thin SiO_2_ sample (thermal SiO_2_):

Elasticity modulus $E_{2}=62.5 GPa=62.5\times{10}^{9} Pa$

Poisson ratio $\gamma_{2}=0.2$

[Data from: Elasticity modulus and Poisson ratio of thermal SiO_2_ reproduced with permission from Khurshudov, A. G., et al. Wear, 203, 22-27 (1997), and Frammelsberger, W., et al. Appl. Surf. Sci., 253, 3615-3626 (2007).]

**Calculation details:**

$$K=\left[ \frac{3}{4}\left( \frac{1-\gamma_{1}^{2}}{E_{1}}+\frac{1-\gamma_{2}^{2}}{E_{2}} \right) \right]^{-1}$$

$$K=\left[ \frac{3}{4}\left( \frac{1-{0.39}^{2}}{170\times{10}^{9}}+\frac{1-{0.2}^{2}}{62.5\times{10}^{9}} \right) \right]^{-1}=65.52\times{10}^{9} Pa$$

Given data: $F_{c}=100 nN=100\times{10}^{-9} N$

The contact force has been calculated by measuring a force distance curve on the surface of the sample, and converting the slope in V/nm into nN/nm, according to the value of the spring constant (*k*) of the tip.

**Condition 1**: $R_{tip}=10.9 nm=10.9 \times{10}^{-9} m$

$$r_{c}=\sqrt[3]{\frac{F_{c}\times R_{tip}}{K}}=\sqrt[3]{\frac{100\times{10}^{-9} N \times10.9 \times{10}^{-9} m}{65.52\times{10}^{9} Pa}}=2.55\times{10}^{-9} m=2.55 nm$$

$$A_{c}=\pi\times r_{c}^{2}=\pi\times({2.55 nm)}^{2}=20.47 {nm}^{2}$$

**Condition 2**: $R_{tip}=25 nm=25 \times{10}^{-9} m$

$$r_{c}=\sqrt[3]{\frac{F_{c}\times R_{tip}}{K}}=\sqrt[3]{\frac{100\times{10}^{-9} N \times25 \times{10}^{-9} m}{65.52\times{10}^{9} Pa}}=3.37\times{10}^{-9} m=3.37 nm$$

$$A_{c}=\pi\times r_{c}^{2}=\pi\times({3.37 nm)}^{2}=35.61 {nm}^{2}$$

**Condition 3**: $R_{tip}=28.5 nm=28.5 \times{10}^{-9} m$

$$r_{c}=\sqrt[3]{\frac{F_{c}\times R_{tip}}{K}}=\sqrt[3]{\frac{100\times{10}^{-9} N \times28.5\times{10}^{-9} m}{65.52\times{10}^{9} Pa}}=3.52\times{10}^{-9} m=3.52 nm$$

$$A_{c}=\pi\times r_{c}^{2}=\pi\times({3.52 nm)}^{2}=38.86 {nm}^{2}$$

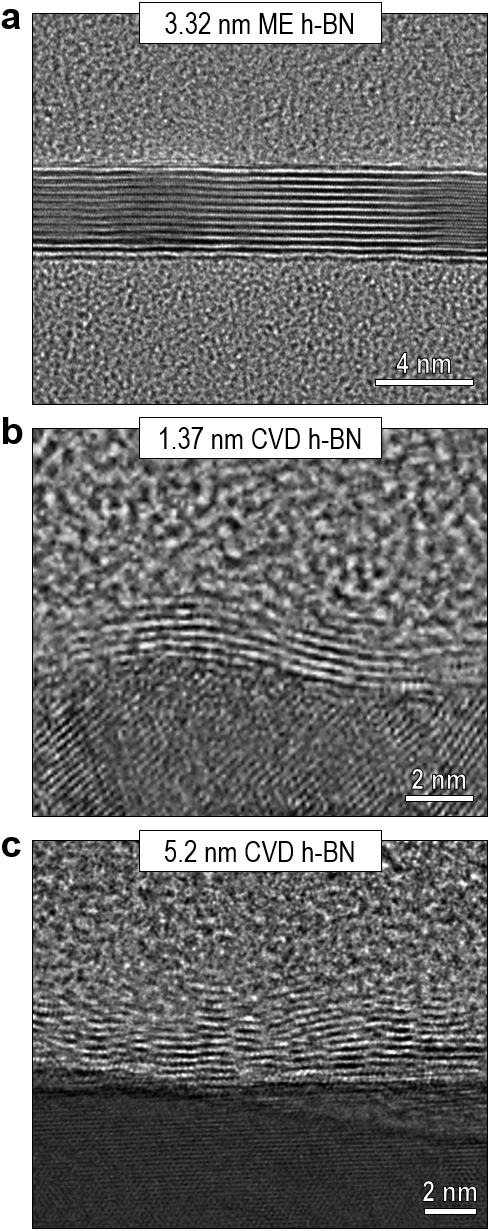


**Figure S16 | High-resolution cross-sectional TEM characterization on h-BN samples. a,** cross-sectional TEM image of a ~3.32 nm mechanically exfoliated h-BN. **b,** cross-sectional TEM image of a ~1.37 nm CVD-grown h-BN. **c,** cross-sectional TEM image of a ~5.2 nm CVD-grown h-BN.


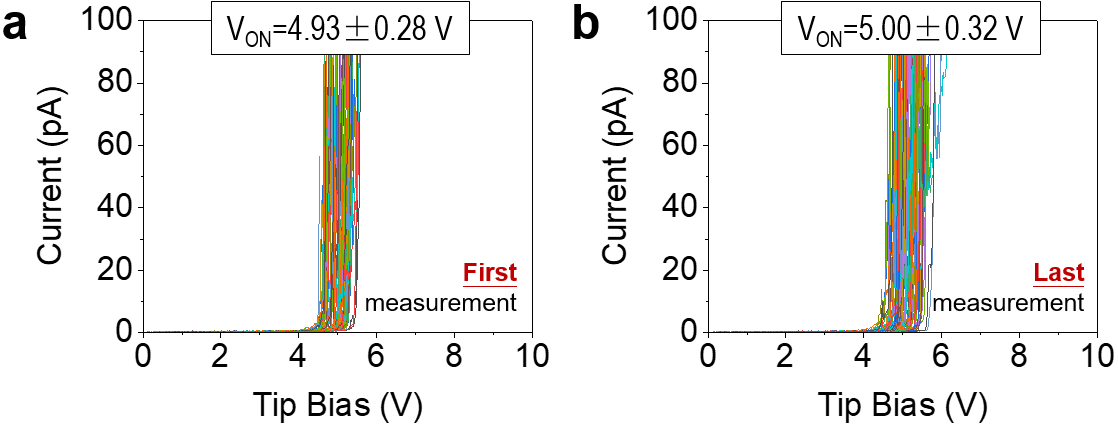


**Figure S17 | CAFM characterization on mechanically exfoliated h-BN sample in air (*RH*=54%) – confirmation of tip condition.** Each *I*-*V* plot contains 100 *I*-*V* curves with a current limitation of 110 pA, and each collected at a different location. **a-b**, The first and the last groups of *I*-*V* curves collected on ~3.32-nm-thick mechanically exfoliated h-BN sample, respectively. **b** is collected after Figure 6**k**.


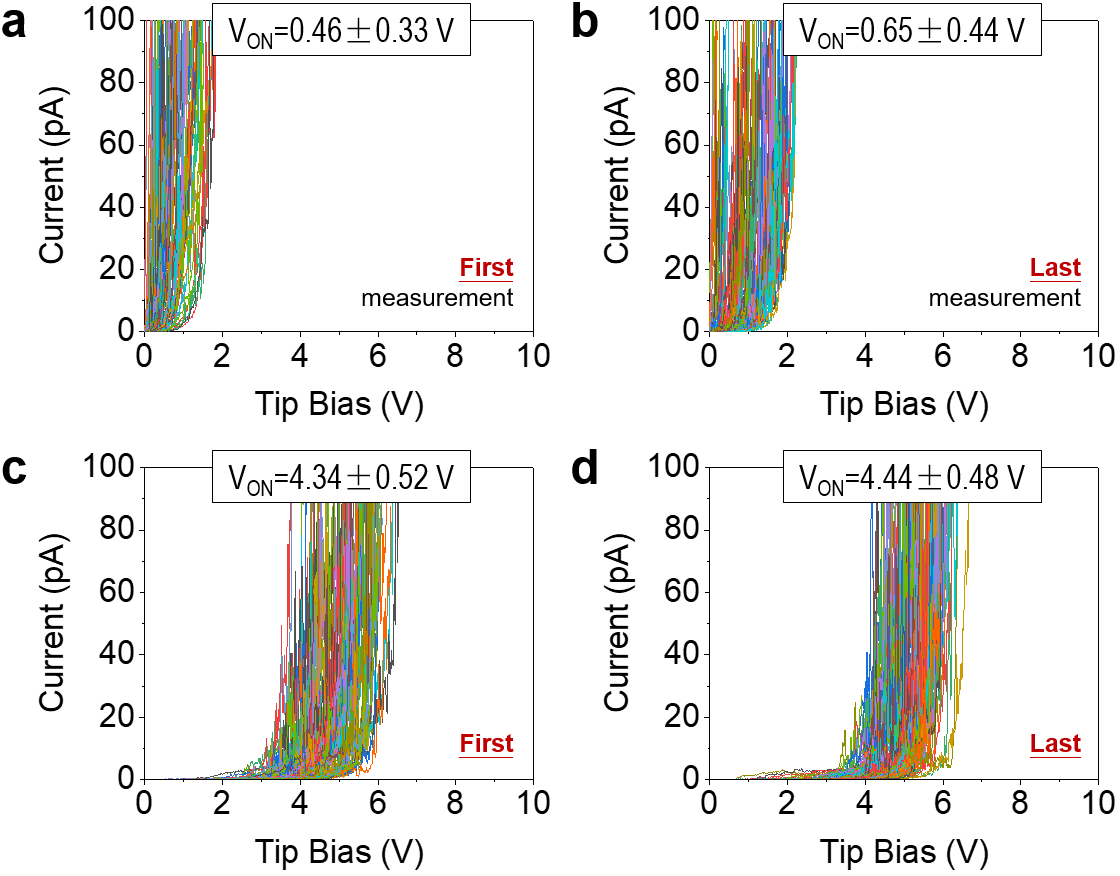


**Figure S18 | CAFM characterization on CVD-grown multilayer h-BN samples in air (*RH*=54%) – confirmation of tip condition.** Each *I*-*V* plot contains 100 *I*-*V* curves with a current limitation of 110 pA, and each collected at a different location. **a-b**, The first and the last groups of *I*-*V* curves collected on ~1.37-nm-thick CVD-grown h-BN sample, respectively. **b** is collected after Figure 7**e**. **c-d**, The first and the last groups of *I*-*V* curves collected on ~5.2-nm-thick CVD-grown h-BN sample, respectively. **d** is collected after Figure 7**f**.


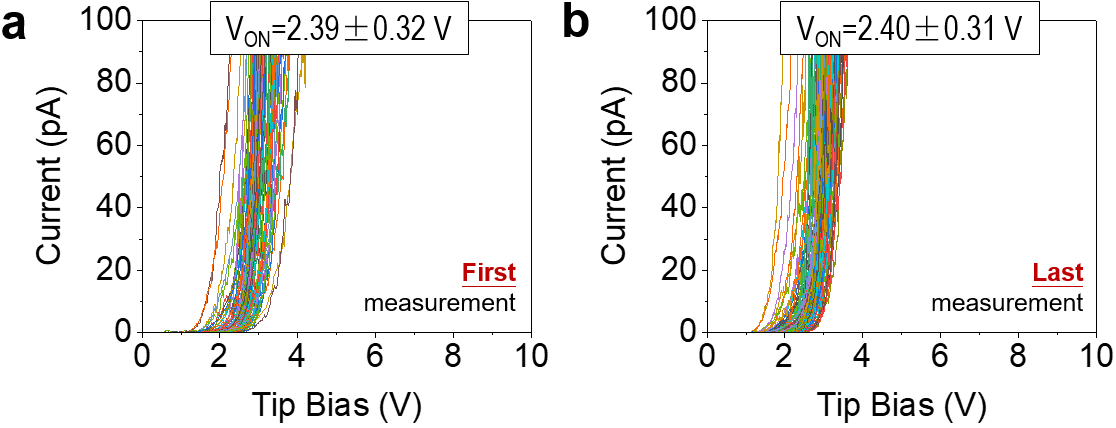


**Figure S19 | CAFM characterization on mechanically exfoliated MoS_2_ sample in air (*RH*=54%) – confirmation of tip condition.** Each *I*-*V* plot contains 100 *I*-*V* curves with a current limitation of 110 pA, and each collected at a different location. **a-b**, The first and the last groups of *I*-*V* curves collected on ~8.66-nm-thick CVD-grown h-BN sample, respectively. **b** is collected after Figure 8**f**.

RH = 54%

RH = 4%

**Figure S20 | Current instabilities occasionally detected at *RH*=4%.** Derivate of the *I*-*V* plots measured in mechanically exfoliated h-BN at relative humidity levels of 54% and 4%.


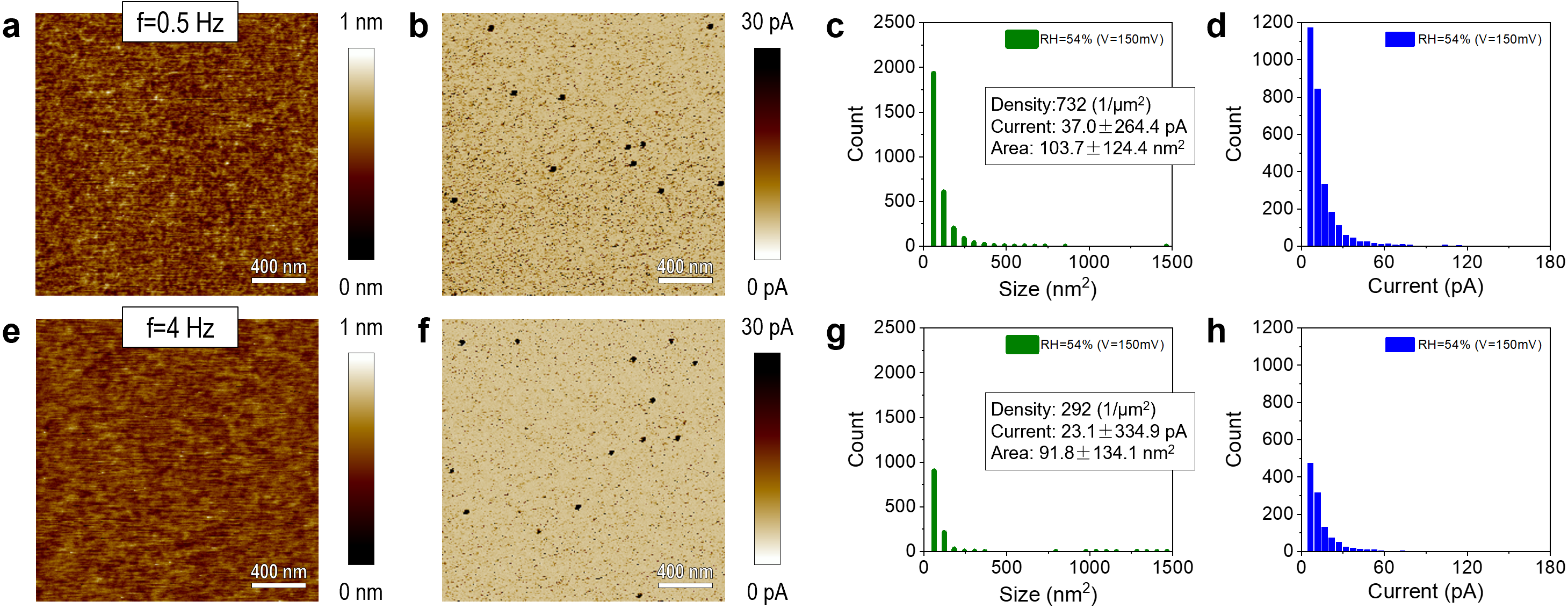


**Figure S21 | Dependence between scan frequency and *A_eff_*.** The sample measured is the same in both cases, 1.5 nm SiO_2_/n^++^Si, meaning that, statistically, the size of the defects is the same. However, when scanning at lower frequencies the spots appear to be bigger and more conductive, even if no other parameter is changed. The reason is simply that the conductive water meniscus surrounding the CAFM tip is more stable and wider at lower scan frequencies. Ultimately, the biggest water meniscus appears when the tip is static, that is, during RVS tests, as explained in the main text.


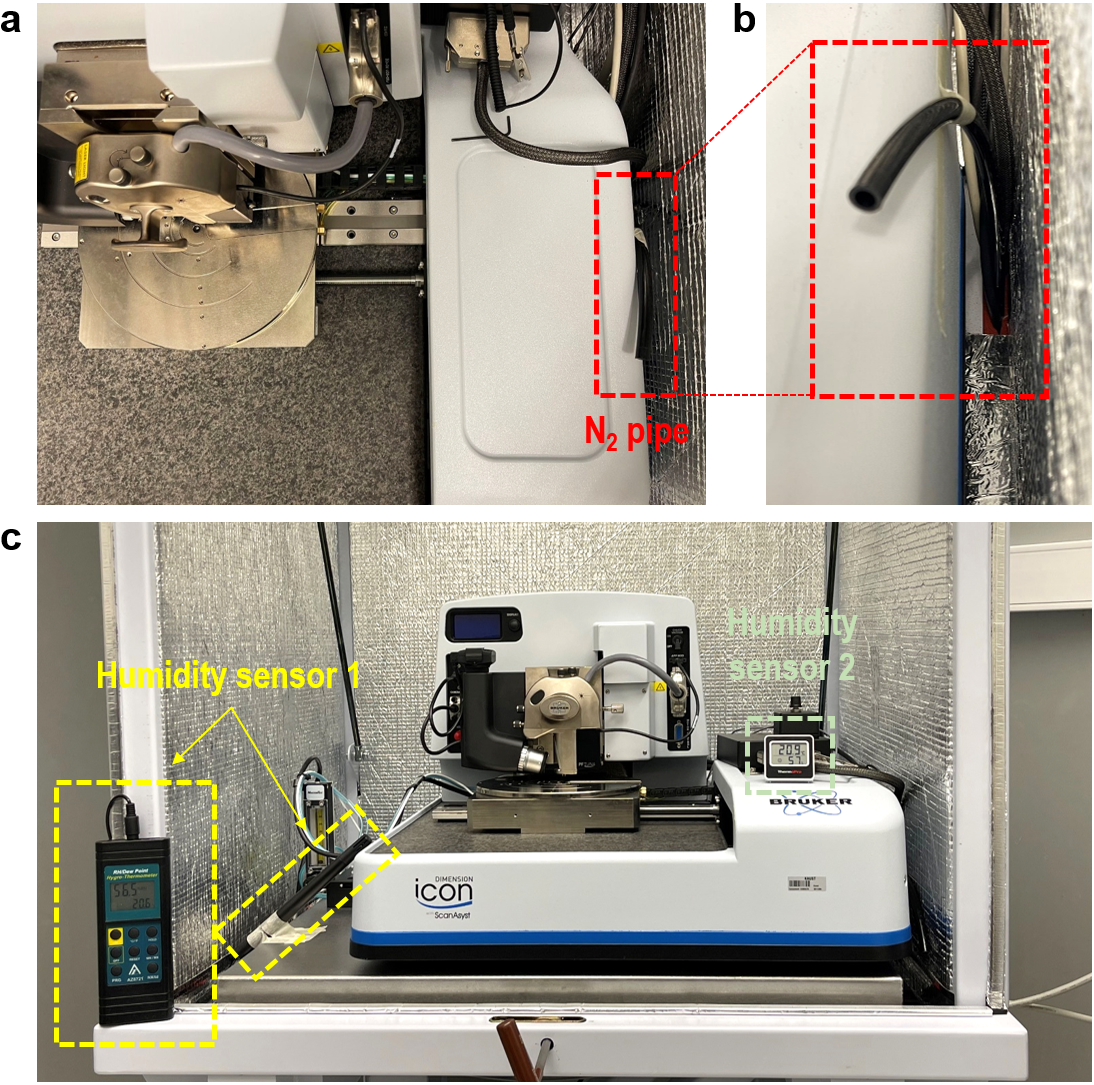


**Figure S22 | Photos of CAFM chamber with environment (humidity) control system. a-b,** Inserted N_2_ pipe at the edge of the CAFM chamber. **c,** two humidity sensors that used to measure the humidity level inside the CAFM chamber.
